# Effects of *PPARD* gene variants on the therapeutic responses to exenatide in chinese patients with type 2 diabetes mellitus

**DOI:** 10.3389/fendo.2022.949990

**Published:** 2022-08-16

**Authors:** Jinfang Song, Na Li, Ruonan Hu, Yanan Yu, Ke Xu, Hongwei Ling, Qian Lu, Tingting Yang, Tao Wang, Xiaoxing Yin

**Affiliations:** ^1^ Jiangsu Key Laboratory of New Drug Research and Clinical Pharmacy, Xuzhou Medical University, Xuzhou, China; ^2^ Department of Pharmacy, Affiliated Hospital of Jiangnan University, Wuxi, China; ^3^ Department of Endocrinology, Affiliated Hospital of Xuzhou Medical University, Xuzhou, China; ^4^ Department of Pharmacy, Affiliated Hospital of Xuzhou Medical University, Xuzhou, China

**Keywords:** *PPARD* gene, genetic variant, type 2 diabetes mellitus, exenatide, insulin resistance

## Abstract

**Background:**

Exenatide is a GLP-1R agonist that often exhibits considerable interindividual variability in therapeutic efficacy. However, there is no evidence about the impact of genetic variants in the *PPARD* on the therapeutic efficacy of exenatide. This research was aimed to explore the influence of *PPARD* gene polymorphism on the therapeutic effect of exenatide, and to identify the potential mechanism futher.

**Methods:**

A total of 300 patients with T2DM and 200 control subjects were enrolled to identify *PPARD* rs2016520 and rs3777744 genotypes. A prospective clinical study was used to collect clinical indicators and peripheral blood of T2DM patients treated with exenatide monotherapy for 6 months. The SNaPshot method was used to identify *PPARD* rs2016520 and rs3777744 genotypes, and then we performed correlation analysis between *PPARD* gene variants and the efficacy of exenatide, and conducted multiple linear regression analysis of factors affecting the therapeutic effect of exenatide. HepG2 cells were incubated with exenatide in the absence or presence of a PPARδ agonist or the siPPARδ plasmid, after which the levels of GLP-1R and the ratio of glucose uptake were determined.

**Results:**

After 6 months exenatide monotherapy, we observed that homeostasis model assessment for insulin resistance (HOMA-IR) levels of the subjects with at least one C allele of the *PPARD* rs2016520 were significantly lower than those with the TT genotype, which suggested that the *PPARD* rs2016520 TT genotype conferred the poor exenatide response through a reduction of insulin resistance, as measured by HOMA-IR. The carriers of G alleles at rs3777744 exhibited higher levels of in waist to hip ratio (WHR), fasting plasma glucose (FPG), hemoglobin A1c (HbA1c) and HOMA-IR compared to individuals with the AA genotype following 6 months of exenatide treatment, potentially accounting for the lower failure rate of exenatide therapy among the AA homozygotes. In an insulin resistant HepG2 cell model, the PPARδ agonists enhanced exenatide efficacy on insulin resistance, with the expression of GLP-1R being up-regulated markedly.

**Conclusion:**

These data suggest that the *PPARD* rs2016520 and rs3777744 polymorphisms are associated with exenatide monotherapy efficacy, due to the pivotal role of PPARδ in regulating insulin resistance through affecting the expression of GLP-1R. This study was registered in the Chinese Clinical Trial Register (No. ChiCTR-CCC13003536).

## Introduction

Within the past few decades, the prevalence of type 2 diabetes mellitus (T2DM) has risen at an astounding rate over the world (1). T2DM is a metabolic disease caused by a complex combination of environmental and genetic factors, characterized by impaired insulin secretion and insulin resistance (2). Genome-wide association studies (GWAS) have led to the identification of hundreds of risk genes, including peroxisome proliferator-activated receptor δ gene (*PPARD*), associated with T2DM susceptibility or abnormal indicators of metabolism (3). *PPARD* is located on chromosome 6p21.1-p21.2, and its coding product PPAR-δ (also named PPAR-β) is a member of the peroxisome proliferator activated receptor family, which is widely distributed in the liver, kidney, cardiac and skeletal muscle, adipose tissue, brain, pancreatic and vasculature (4). *PPARD* was not observed as the susceptibility gene for T2DM in a case-control clinical study conducted in a Korean population in 2004, but *PPARD* variants were founded to be associated with elevated fasting plasma glucose (FPG) and body mass index (BMI) (5). Studies in Chinese Han population have shown that *PPARD* rs2016520 variant (also named +294T > C or -87T > C) is associated with blood glucose, insulin level and insulin resistance, and is a key factor affecting the development of metabolic syndrome and T2DM (6, 7). Studies in Mexican population have produced similar results (8). Meanwhile, pathogenesis research already pointed that PPAR-δ plays an important role in insulin resistance and islet β-cell function (9–11). More recently, PPAR-δ activation came into focus as an interesting novel approach for the treatment of metabolic syndrome. Both preclinical and clinical studies showed that PPAR-δ specific agonist therapy enhanced β-oxidation, decreased free fatty acid, and improved insulin sensitivity (12, 13). Current studies have demonstrated the effect of *PPARD* on metabolic metrics, but the mechanisms responsible for this effect are not well characterized.

Exenatide is a glucagon-like peptide 1 (GLP1) analogue that exerts its pharmacological effects through activating glucagon-like peptide 1 receptor (GLP1-R). It mirrors many of the effects of GLP-1 and improve glycemic control through a combination of mechanisms, which includes glucose-dependent stimulation of insulin secretion, suppression of glucagon secretion, slowing of gastric emptying and reduction of appetite (14, 15). The results of a multi-center randomized controlled clinical trial showed that there were significant individual differences in glycemic control, islet function, and body mass index in T2DM patients undergoing exenatide monotherapy for 48 weeks (16). Our research team conducted a previous retrospective clinical study to investigate the hypoglycemic efficacy of 148 T2DM patients treated with exenatide and discovered that the proportion of T2DM patients who did not respond to exenatide treatment was as high as 37.84% (17). The non-response of exenatide treatment not only affects the glycemic standard of T2DM patients and reduces the medication compliance, but also brings a heavy economic burden to patients. Some studies have shown that gene variants can affect the stimulatory effect of GLP-1 receptor agonists on insulin secretion (18–20). The TCF7L2 rs7903146 mutation was found to attenuate GLP-1-induced insulin secretion in German and Danish populations (18, 19). Another study suggested that mutations in the WFS1 gene attenuated GLP-1-induced insulin secretion, but not in relation with insulin sensitivity (20). Therefore, genetic factors are essential to the efficacy of exenatide. We analyzed the GLP-1R promoter using the National Center for Biotechnology Information (https://www.ncbi.nlm.nih.gov/) and JASPAR database (http://jaspar.genereg.net/), and found the potential PPARδ binding site (**Supplementary Figure S1**).

Pharmacogenomic studies have identified genetic factors as an important cause of individual differences in the efficacy of hypoglycemic drugs (2, 21, 22). However, it remains unknown whether *PPARD* single nucleotide polymorphisms (SNPs) have the same influence on the therapeutic effects of exenatide. In this study, we investigated the association between *PPARD* variants and the efficacy of exenatide in newly diagnosed Chinese T2DM patients who received exenatide monotherapy for 6 months, and further explored the potential mechanism.

## Materials and methods

### Participants and study design

A total of 300 patients with T2DM (196 male and 104 female) and 200 healthy controls (139 male and 61 female) were enrolled for *PPARD* variants analysis. T2DM patients and healthy subjects were recruited from the Department of Endocrinology and Health Screening Center, Affiliated Hospital of Xuzhou Medical University, Xuzhou, China. T2DM was diagnosed according to the 1999 World Health Organization criteria. The inclusion criteria were: newly diagnosed T2DM without drug therapy; 25 to 70 years old; Hemoglobin A1c (HbA1c) 7%-12%; BMI 20-35 kg/m^2^; Stable body weight (≤10% change within 3 months); Female subjects were required to use birth control pills during the three months prior to screening and during the study, or surgical contraception, or postmenopausal women. Subjects with acute or severe chronic diabetic complications, serious comorbid diseases, New York Heart Function Scale (NYHA) III~IV, severe osteoporosis or a history of fractures, alanine aminotransferase (ALT) and aspartate transaminase (AST) ≥2.5 times of upper limit, or serum creatinine level ≥133 µmol/L, severe gastrointestinal dysfunction, ongoing use of weight-loss drugs, glucocorticoids, drugs that affecting gastrointestinal motility, transplant therapy drugs, any investigational drugs, a history of pancreatitis, or serum triglyceride level ≥5 mmol/L were excluded. A total of 105 newly diagnosed T2DM patients (73 male and 32 female) with different *PPARD* rs2016520 and rs3777744 genotypes were randomly selected to receive exenatide injection subcutaneously for 6 months and completed the follow-up. During the 1st week to the 4th week, exenatide was given 5 µg once, twice a day, after which the dose was adjusted to 10 µg once, twice a day. The protocol was approved by the Ethics Committee of the Affiliated Hospital of Xuzhou Medical University. Written informed consent was obtained from each participant before taking part in the study.

### Anthropometric and biochemical measurements

The general anthropometric parameters such as height, weight, waist circumference and hip circumference were measured in the morning on an empty stomach. Waist circumference was measured at the midpoint of the line connecting the lower rib cage and the skeleton, and hip circumference was measured at the level of the greater trochanter of the femur. BMI and waist to hip ratio (WHR) were calculated. BMI = body weight (kg)/height (m)^2^, WHR = waist circumference (cm)/hip circumference (cm). Venous blood was collected both after fasting overnight and 2 h later during a standard oral glucose tolerance test. Parameters were measured before administration of exenatide, 3 months and 6 months after administration. Plasma glucose and serum lipids were detected using a Roche Cobas8000 analyzer (Roche, Basel, Switzerland) with standard laboratory methods. Accordingly, the levels of insulin and HbA1c were measured by an electrochemiluminescence assay (Roche, Shanghai, China) and high-performance liquid chromatography (HPLC). The homeostasis model assessment for insulin resistance (HOMA-IR) and homeostasis model assessment for beta cell function (HOMA-B) were calculated using the formula: HOMA-IR = fasting insulin (mU/L)×fasting plasma glucose (mmol/L)/22.5; homeostasis model assessment HOMA-B = 20×fasting serum insulin (FINS)/(FPG-3.5) (23).

### Genotyping

Genomic DNA was extracted from peripheral whole blood leukocytes with a DNA extraction kit (Tiangen Biotech, Beijing, China) according to the manufacturer’s protocol. The allelic discrimination of *PPARD* rs2016520 and rs3777744 was performed by SNaPshot assay (Genesky Biotechnologies Inc., Shanghai, China) with the standard protocol (24).

### Definition of the response to exenatide

The guideline of National Institute for Health and Care Excellence (NICE) define T2DM patients who have a ≥1.0% reduction in HbA1c or a ≥3% reduction in body weight after 6 months of treatment with GLP-1 agonists as the treatment response group, and those who do not meet these criteria are defined as Non-responders (25). Based on UK prospective diabetes study (UKPDS)results, a 1% decrease in HbA1c was associated with a 37% reduction in the risk of diabetic microvascular complications and a 21% reduction in diabetes-related end points (26). Since diabetes complications are closely related to HbA1c level, this study mainly evaluated HbA1c changes after exenatide treatment, and defined those T2DM patients whose HbA1c decreased ≥1.0% or endpoint HbA1c < 7.0% after 6 months of exenatide treatment as Responders. Those who do not meet the above criteria are Non-responders.

### Cell culture

The human hepatoma cell line (HepG2) was obtained from the Type Culture Collection of the Chinese Academy of Sciences (Shanghai, China), cultured in Dulbecco’s modified Eagle’s medium supplemented with 10% fetal bovine serum (Gibco BRL, USA) and were placed in a humidified incubator containing an atmosphere of 5% CO_2_ at 37°C. The insulin-resistant cell model was induced using the previous method (27). HepG2 cells were allowed to attach for 12 h and then serum-starved for 8 h. HepG2 cells were incubated with fresh medium containing 1% FBS, 10^-3^ mmol/L insulin (Wanbang Biologic & Medicinal Co., Ltd., Jiangsu, China) for 6 h. Subsequently, the medium was exchanged with medium containing 1% FBS, 10^-6^ mmol/L insulin and Exendin-4 (10^-1^ mmol/L, Sigma). Cells were incubated in this medium for 12 h.

### Gene transfection

The transfection of plasmids (6 µg) was carried out using Lipofectamine 3000 Transfection Reagent (Invitrogen, USA) according to the manufacturer’s instructions. The ratio of plasmids (μg) and transfection reagents (μL) was 3:4 (27–29). The cells were harvested 48 h after transfection.

### Glucose uptake tests of HepG2 cells

Glucose content was determined by the enzymatic method of the diagnostic kit using 10 μL of medium (Nanjing Jiancheng Bioengineering Inst, China). Data were expressed as extracellular glucose consumption (nmol/mg protein) and calculated as follows: [before extracellular glucose content (nmol) - after extracellular glucose content (nmol)]/mg cellular protein (27), which was measured using the Nanodrop 2000 spectrophotometer (Thermo, USA).

### Western blot analysis for protein levels

Western blot analysis was performed as previously described (28), and the antibodies were applied at concentrations according to the manufacturer’s instructions. Actin served as the loading control. Bands were quantified using Image J software. Anti-Actin (AP0060, Bioworld, USA), anti-PPARδ (101562-AP, Proteintech, USA) and anti-GLP-1R (DF7750, Affinity Biosciences, USA) were used in our study.

### Real-time quantitative RT-PCR

Total RNA in HepG2 cells was isolated using Trizol Reagent (15596-026, Invitrogen, USA) according to the manufacturer’s instructions, and the steps of RT-PCR were carried out as described previously (29, 30). Data were normalized to internal control β-actin mRNA. Primers were designed and synthesized by Sangon Biotech (Shanghai, China). We used the primer pair 5′-TCTGGAATGGTCTGGAGTGGTCTG-3′ (forward) and 5′-GCCTTGAAGCAGTCCTGTAGAGATC-3′ (reverse) for human PPARδ, 5′- GCAAAGACCTGTACGCCAAC -3′ (forward) and 5′- AGTACTTGCGCTCAGGAGGA -3′ (reverse) for human β-actin, 5′-CCTCCAGATGTCCCCTCCAGATG-3′ (forward) and 5′-CTAAGTGTGCCGCTGCTCCTTC-3′ (reverse) for mouse PPARδ, 5′-AGAGGGAAATCGTGCGTGAC-3′ (forward) and 5′-CAATAGTGATGACCTGGCCGT-3′ (reverse) for mouse β-actin, 5′-GTTCCCCTGCTGTTTGTTGT-3′ (forward) and 5′-CTTGGCAAGTCTGCATTTGA-3′ (reverse) for human GLP-1R.

### Statistical analysis

All data were expressed as mean ± standard deviation (Mean ± SD) or percentage as appropriate. Statistical analyses were performed using SPSS software (version 13.0 for Windows; SPSS Inc., Chicago, IL, USA). Chi-square test was used to compare the Hardy-Weinberg equilibrium, allele frequency and genotype distribution among different groups. Linkage disequilibrium (LD) among SNPs was estimated in subjects using Haploview version 3.2. The two-sample t-test was used to compare the baseline characteristics between T2DM patients and healthy subjects. The paired Student’s t-test was applied for evaluating the parameters between the two groups before and after exenatide treatment. For parameters of normal distribution, two-sample t-test was used for comparison between two groups, and one-way analysis of variance (ANOVA) was used for comparison among multiple groups. Parameters with nonnormal distribution were analyzed by the Mann-Whitney U-test or the Kruskal-Wallis test. ANOVA for repeated measurement was used to compare the parameters collected at different treatment time points of the same patient. Statistical power was calculated by power calculator software (http://www.ncss.com). In the experimental study, differences between treated and control results were compared using one-way ANOVA with a Tukey-Kramer post-test for multiple comparisons or unpaired t-test. Two-sided tests were used for all analyses, and *P* < 0.05 indicated statistically significant.

## Results

### Allelic frequency analysis

In the present study, *PPARD* rs2016520 and rs3777744 variants were genotyped in 300 patients with T2DM (196 male and 104 female) and 200 healthy controls (139 male and 61 female) (**Table 1**). The frequency of the A allele at the *PPARD* rs3777744 locus was lower in patients with T2DM than in healthy controls (57.00% vs. 62.75%, *P* = 0.004), whereas the *PPARD* rs2016520 variant, there was no significant difference in allele frequencies between the two groups. The genotype distributions of rs2016520 (*P* = 0.105) and rs3777744 (*P* = 0.171) SNPs were in Hardy-Weinberg equilibrium. Assessment of the LD between the variants using our control subjects revealed a relatively low disequilibrium between rs2016520 and rs3777744 (D’ = 0.110).

**Table 1:** Comparison of genotype and frequencies of *PPARD* variants between healthy controls (n = 200) and patients with type 2 diabetes mellitus (T2DM) (n = 300).

Genotypes	Healthy subjects (frequency, %)	T2DM patients (frequency, %)	*P* value
*PPARD* rs2016520			
TT	106 (53.00)	148 (49.33)	
TC	83 (41.50)	125 (41.67)	
CC	11 (5.50)	27 (9.00)	0.324
*Alleles*			
T	295 (73.75)	421 (70.17)	
C	105 (26.25)	179 (29.83)	0.565
*PPARD* rs3777744			
AA	84 (42.00)	95 (31.67)	
AG	95 (47.50)	150 (50.67)	
GG	21 (10.50)	55 (17.67)	0.014
*Alleles*			
A	263 (62.75)	340 (57.00)	
G	137 (37.25)	260 (43.00)	0.004

### Assessment of clinical characteristics in T2DM patients with different rs2016520 and rs3777744 genotypes

In the present study, the baseline clinical characteristics of 300 T2DM patients with different rs2016520 and rs3777744 genotypes were analyzed (**Tables 2**, **3**). There were no significant differences in gender, age, postprandial plasma glucose (PPG), HbA1c, postprandial serum insulin (PINS), HOMA-B, triglyceride (TG), high-density lipoprotein-cholesterol (HDL-C) and low-density lipoprotein-cholesterol (LDL-C) between different genotype groups. However, significant differences were observed among patients with different genotypes of *PPARD* rs2016520 in terms of BMI (*P* = 0.000), WHR (*P* = 0.001) and TG (*P* = 0.006) (**Supplementary Figure S2**). Compared to patients with the AA genotype, patients with the rs3777744 risk G allele had noticeably higher WHR (*P* = 0.000), PPG (*P* = 0.011), FINS (*P* = 0.004) and HOMA-IR (*P* = 0.000) levels (**Supplementary Figure S3**).

**Table 2:** The baseline characteristics in type 2 diabetes mellitus (T2DM) patients with various *PPARD* rs2016520 genotypes before treatment with exenatide (n=300).

Parameters	*PPARD* rs2016520 genotype			Overall *P* value	Adjusted *P* value		
	TT	TC	CC		TT to TC	TC to CC	TT to CC
N (male/female)	148 (96/52)	125 (80/45)	27 (20/7)	0.600			
Age (years)	49.68 ± 12.88	50.08 ± 12.88	48.04 ± 14.27	0.760	1.000	1.000	1.000
BMI (kg/m^2^)	27.16 ± 2.67	28.35 ± 4.10	29.69 ± 3.80	0.000	0.014	0.195	0.001
WHR	0.93 ± 0.06	0.96 ± 0.07	0.98 ± 0.08	0.001	0.014	0.488	0.007
FPG (mmol/L)	9.68 ± 2.54	10.21 ± 2.55	10.62 ± 2.31	0.091	0.254	1.000	0.233
PPG (mmol/L)	14.78 ± 3.86	15.70 ± 4.30	16.06 ± 3.53	0.099	0.182	1.000	0.388
HbA1c (%)	9.09 ± 1.63	9.28 ± 1.76	9.42 ± 1.71	0.508	1.000	1.000	1.000
FINS (mU/L)	12.81 ± 8.28	14.32 ± 12.37	11.53 ± 7.04	0.296	0.659	0.585	1.000
PINS (mU/L)	43.59 ± 32.83	42.53 ± 45.55	33.48 ± 15.79	0.439	1.000	0.604	0.779
HOMA-IR	5.39 ± 3.97	6.27 ± 5.09	5.65 ± 4.15	0.269	0.321	1.000	1.000
HOMA-B	52.70 ± 51.27	55.49 ± 77.35	33.70 ± 18.49	0.251	1.000	0.294	0.430
TG (mmol/L)	2.60 ± 1.86	2.17 ± 1.53	3.42 ± 3.27	0.006	0.200	0.007	0.125
TC (mmol/L)	5.13 ± 1.27	5.03 ± 1.39	5.29 ± 1.60	0.626	1.000	1.000	1.000
HDL-C (mmol/L)	1.10 ± 0.30	1.12 ± 0.75	1.02 ± 0.20	0.681	1.000	1.000	1.000
LDL-C (mmol/L)	3.01 ± 0.88	2.96 ± 0.97	2.88 ± 0.92	0.771	1.000	1.000	1.000

BMI, body mass index; WHR, waist to hip ratio; FPG, fasting plasma glucose; PPG, postprandial plasma glucose; HbA_1c_, hemoglobin A_1c_; FINS, fasting serum insulin; PINS, postprandial serum insulin; HOMA-IR, homeostasis model assessment for insulin resistance; HOMA-B, homeostasis model assessment for beta cell function; TG, triglyceride; TC, total cholesterol; HDL-C, high-density lipoprotein-cholesterol; and LDL-C, low-density lipoprotein-cholesterol.

**Table 3:** The baseline characteristics in type 2 diabetes mellitus (T2DM) patients with various *PPARD* rs3777744 genotypes before treatment with exenatide (n=300).

Parameters	*PPARD* rs3777744 genotype			Overall *P* value	Adjusted *P* value		
	AA	AG	GG		AA to AG	AG to GG	AA to GG
N (male/female)	95 (66/29)	150 (92/58)	55 (38/17)	0.346			
Age (years)	50.46 ± 13.46	50.37 ± 12.77	46.55 ± 12.40	0.136	1.000	0.184	0.224
BMI (kg/m^2^)	27.52 ± 3.10	27.94 ± 3.47	28.36 ± 4.26	0.355	1.000	1.000	0.474
WHR	0.88 ± 0.06	0.94 ± 0.07	0.95 ± 0.07	0.000	0.000	1.000	0.000
FPG (mmol/L)	9.76 ± 2.26	10.16 ± 2.71	9.92 ± 2.53	0.483	0.237	0.547	0.722
PPG (mmol/L)	13.82 ± 2.87	15.22 ± 3.26	15.38 ± 4.08	0.011	0.018	1.000	0.052
HbA1c (%)	9.08 ± 1.62	9.17 ± 1.58	9.51 ± 2.06	0.310	1.000	0.592	0.417
FINS (mU/L)	11.02 ± 5.81	13.58 ± 10.45	16.59 ± 13.64	0.004	0.152	0.170	0.003
PINS (mU/L)	36.60 ± 27.02	45.36 ± 44.24	43.46 ± 33.29	0.230	1.000	1.000	0.848
HOMA-IR	4.84 ± 2.52	5.94 ± 3.62	6.84 ± 4.98	0.000	0.021	0.119	0.001
HOMA-B	43.60 ± 51.32	51.32 ± 52.57	69.16 ± 98.61	0.050	1.000	0.201	0.045
TG (mmol/L)	2.75 ± 2.33	2.36 ± 1.64	2.43 ± 1.92	0.306	0.394	1.000	0.994
TC (mmol/L)	5.09 ± 1.35	5.16 ± 1.47	4.99 ± 0.99	0.720	1.000	1.000	1.000
HDL-C (mmol/L)	1.06 ± 0.24	1.09 ± 0.32	1.18 ± 1.09	0.340	1.000	1.000	0.440
LDL-C (mmol/L)	2.96 ± 0.89	2.98 ± 0.96	2.99 ± 0.87	0.969	1.000	1.000	1.000

BMI, body mass index; WHR, waist to hip ratio; FPG, fasting plasma glucose; PPG, postprandial plasma glucose; HbA_1c_, hemoglobin A_1c_; FINS, fasting serum insulin; PINS, postprandial serum insulin; HOMA-IR, homeostasis model assessment for insulin resistance; HOMA-B, homeostasis model assessment for beta cell function; TG, triglyceride; TC, total cholesterol; HDL-C, high-density lipoprotein-cholesterol; and LDL-C, low-density lipoprotein-cholesterol.

### Effects of the rs2016520 and rs3777744 variants on the efficacy of exenatide in T2DM patients

After 6 months of exenatide treatment, the BMI, WHR, FPG, PPG, HbA1c, HOMA-IR, total cholesterol (TC), TG, and LDL-C values of patients with T2DM were significantly decreased, but the levels of FINS and HOMA-B increased, compared with the pretreatment values (**Supplementary Table S1**).

**Figure 1:**
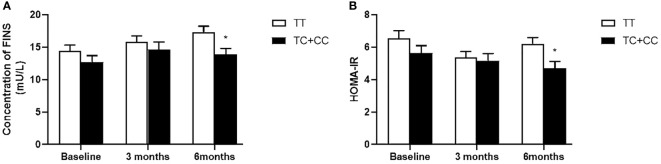
Comparison of fasting serum insulin (FINS) **(A)** and homeostasis model assessment for insulin resistance (HOMA-IR) **(B)** between T2DM patients with the TT genotype (n = 62) and those with TC and CC genotypes (n = 43) genotypes of *PPARD* rs2016520 in T2DM patients before, at 3 months and at 6 months of exenatide treatment. Data are expressed as the mean ± SE, *P<0.05 compared with TT genotype group (n= 105).

**Figure 2:**
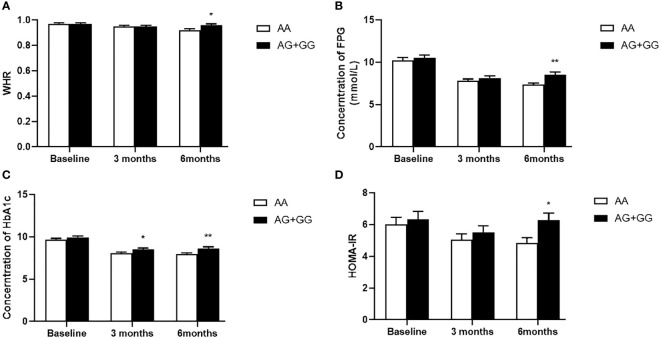
Comparison of waist to hip ratio (WHR) **(A)**, fasting plasma glucose (FPG) **(B)**, hemoglobin A1c (HbA1c) **(C)** and homeostasis model assessment for insulin resistance (HOMA-IR) **(D)** between T2DM patients with the AA genotype (n = 50) and those with AG and GG genotypes (n = 55) genotypes of *PPARD* rs3777744 in T2DM patients before, at 3 months and at 6 months of exenatide treatment. Data are expressed as the mean ± SE, *P<0.05 and **P<0.01 compared with AA genotype group (n = 105).

Our data also showed that patients with genotype TT at *PPARD* rs2016520 had poor efficacy of exenatide monotherapy with respect to HOMA-IR than C allele carriers (**Supplementary Table S2** and **Figure 1**). Moreover, patients with *PPARD* rs3777744 AG + GG genotypes had attenuated efficacy of exenatide monotherapy with respect to WHR, FPG, HbA1c and HOMA-IR compared with AA genotype carriers (**Supplementary Table S2** and **Figure 2**).

### Association of the rs2016520 and rs3777744 variants with response rate to exenatide treatment

In order to assess the association of the *PPARD* genetic variants with the response rate to exenatide treatment in the present study, the genotypes and allele frequencies were analyzed in the Responder and Non-responder groups (**Table 4**). According to predetermined exenatide response criteria, *PPARD* rs3777744 A allele carriers exhibited higher response rate to exenatide treatment (*P* = 0.007); AA allele homozygotes had the highest response rate (84.00%), while AG heterozygous and GG homozygous had 61.36% and 54.55%, respectively (*P* = 0.022). No significant effects of *PPARD* rs2016520 variant on exenatide therapy were observed.

**Table 4:** Genotype and allele distributions between responders and non-responders of *PPARD* rs2016520 and rs3777744 variants (n = 105).

	Genotype			*P* value	Allele frequency		*P* value
*PPARD* rs2016520	TT	TC	CC		T	C	
Responder (%)	42 (67.74%)	25 (75.75%)	8 (80.00%)		109 (69.43%)	41 (77.36%)	
Non-responder (%)	20 (32.26%)	8 (24.24%)	2 (20.00%)	0.584	48 (30.57%)	12 (22.64%)	0.182
*PPARD* rs3777744	AA	AG	GG		A	G	
Responder (%)	42 (84.00%)	27 (61.36%)	6 (54.55%)		111 (77.08%)	39 (59.09%)	
Non-responder (%)	8 (16.00%)	17 (38.64%)	5 (45.45%)	0.022	33 (22.92%)	27 (40.91%)	0.007

To further determine the correlation between *PPARD* variant and improvement in HbA1c after exenatide treatment, a multiple linear regression model was used, with the dependent variable being the decrease in HbA1c after 6 months of exenatide treatment and the independent variables being age, gender, baseline BMI, baseline WHR, baseline HbA1c, rs2016520 and rs3777744. The results showed that the improvement of HbA1c after exenatide treatment was significantly correlated with baseline HbA1c and rs3777744, and the difference between *PPARD* rs3777744 AG+GG genotype and AA genotype in the improvement of HbA1c was statistically significant (*P* = 0.009). Higher the baseline HbA1c was, more significantly the HbA1c improved after 6 months of exenatide treatment (*P* = 0.000) (**Table 5**).

**Table 5:** Multiple linear regression analysis of HbA1c improvement after 6 months of exenatide treatment (n = 105).

Variables	β	95% CI	*P* value
Age (years)	0.011	(-0.003, 0.024)	0.630
Sex (male/female)	0.096	(-0.006, -0.023)	0.261
Baseline BMI (kg/m^2^)	0.045	(-0.025, 0.114)	0.205
Baseline WHR	0.709	(-3.477, 4.865)	0.736
Baseline HbA1c (%)	-0.553	(-0.705, -0.402)	0.000
rs2016520	0.196	(-0.155, 0.546)	0.271
rs3777744	0.432	(0.233, 1.321)	0.009

BM, body mass index; WHR, waist to hip ratio; HbA_1c_, hemoglobin A_1c_.

### Expression of PPARδ in liver tissues of *db/db* mice and in an insulin-resistant HepG2 cell model

To clarify the effect of insulin resistance on the expression level of PPARδ, we used RT-PCR and Western blot to detect the expression level of PPARδ in the liver tissues of *db/db* mice and in an insulin-resistant HepG2 cell model. When compared with the *db/m* group, the mRNA and protein levels of PPARδ in the liver tissues of *db/db* group were significantly lower (*P* < 0.05) (**Supplementary Figures S4A, B**). The results of *in vitro* experiments showed that in HepG2 cells, there was no difference between PPARδ expression in the solvent control group (SC) and the negative control group (NC), but both mRNA and protein expression of PPARδ were significantly lower in the insulin resistant group (IR) (*P* < 0.05) (**Supplementary Figures S4C, D**). According to the *in vivo* and *in vitro* results, PPARδ expression levels were significantly decreased in insulin resistant HepG2 cells and in liver tissue of *db/db* mice.

### PPARδ controls exenatide therapeutic efficacy in insulin resistance by regulating the expression of GLP-1R

To further validate the mechanism by which PPARδ regulates exenatide therapeutic efficacy, relationship between PPARδ activity and GLP-1R expression was investigated. The silencing PPARδ plasmid and silencing negative control (siNC) plasmid were transfected in HepG2 cells, and the knockdown efficiency of the three plasmids (siPPARδ-1, siPPARδ-2 and siPPARδ-3) was detected. The RT-PCR and western blot results showed the most significant reduction in the siPPARδ-3 group (**Supplementary Figure S5**). Therefore, the silencing plasmid named siPPARδ-3 was finally selected for the follow-up experiment.

**Figure 3:**
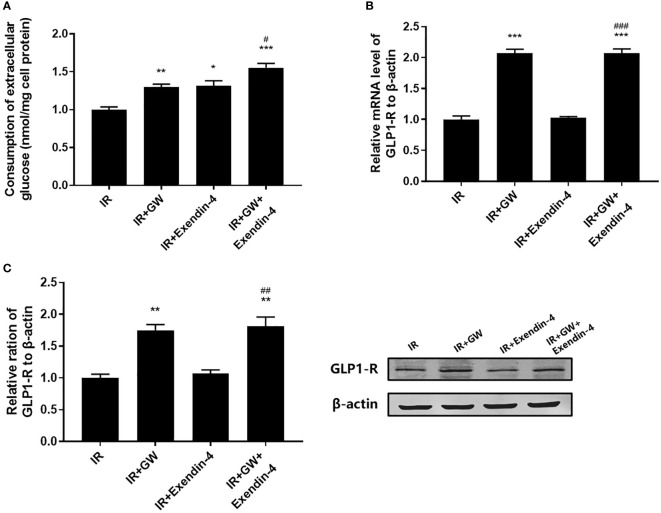
Effect of PPARδ agonists (GW501516) on the exenatide therapeutic efficacy by regulating the expression of GLP-1R. **(A)** Enzymatic methods were used to assay for glucose. **(B)** The mRNA level of GLP-1R was measured by RT-PCR. **(C)** The relative protein expression level of GLP-1R was measured by Western blot. Data are expressed as the mean ± SE, n = 3. ^*^ *P* < 0.05, ^**^ *P* < 0.01 and ^***^ *P* < 0.001 compared with IR. ^#^ *P* < 0.05, ^##^ *P* < 0.01 and ^###^ *P* < 0.001 compared with IR+Exendin-4.

**Figure 4:**
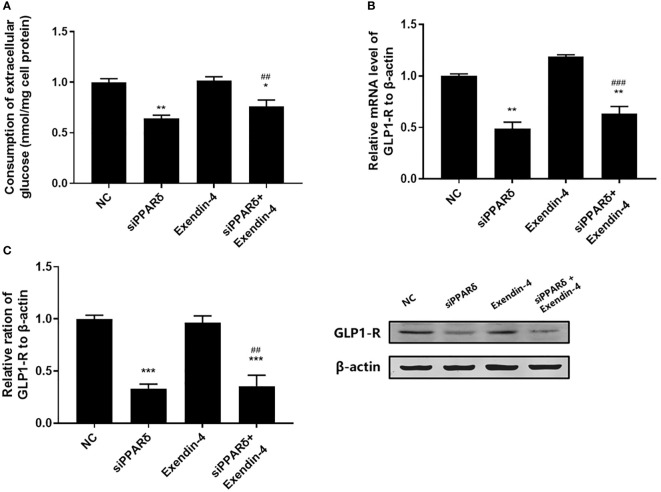
Effect of PPARδ knockdown on the exenatide therapeutic efficacy by regulating the expression of GLP-1R. **(A)** Enzymatic methods were used to assay for glucose. **(B)** The mRNA level of GLP-1R was measured by RT-PCR. **(C)** The relative protein expression level of GLP-1R was measured by Western blot. Data are expressed as the mean ± SE, n = 3. ^*^ *P* < 0.05, ^**^ *P* < 0.01 and ^***^ *P* < 0.001 compared with NC. ^##^ *P* < 0.01, ^###^ *P* < 0.001 compared with Exendin-4.

As shown in **Figure 3A**, both PPARδ agonists (GW501516) and Exendin-4 significantly increased the ratio of glucose uptake in IR HepG2 cells, and the efficacy of Exendin-4 was significantly enhanced after treatment with GW501516. In addition, the expression of GLP-1R in IR HepG2 cells were up-regulated markedly after administration of PPARδ agonist (**Figure 3B, C**). As predicted, the knockdown of PPARδ hindered glucose metabolism and down-regulated the expression of GLP-1R in HepG2 cells (**Figure 4**). These results indicated that PPARδ plays a pivotal role in insulin resistance through regulating the expression of GLP1-R and influences the ability of exenatide to agonize GLP-1R to improve insulin resistance.

## Discussion

In the current study, we evaluated the potential impact of two SNPs (rs2016520 and rs3777744) of *PPARD* on the outcomes of exenatide in treating Chinese patients with T2DM. Genetic variants associated with T2DM susceptibility or metabolism-related indicators have been proved in several pharmacogenomic studies to be important factors in the efficacy of exenatide (18–20). The results of this study revealed that patients with the TT genotype of *PPARD* rs2016520 or the G allele of rs3777744 may have a weaker response to exenatide therapy, indicating that the *PPARD* genotype can be used as a predictor of response to exenatide. Therefore, our findings support the prior generation of genotyping and screening of patients with T2DM for gene-directed individualized dosing in the clinical application of exenatide. Moreover, we also observed the critical role of PPARδ in regulation of the expression of GLP-1R, the receptor for exenatide, and effects on insulin resistance.

Common SNPs in *PPARD* are associated with an increased risk of impaired glucose tolerance, fasting glucose elevation and insulin resistance in populations with diverse ethnic backgrounds including Chinese, Korean and Mexican (5–8). In the present study, we focused on genetic variants in PPARD and found that the risk G allele of rs3777744 (43.00%) in patients with T2DM had a higher frequency than that in the control group (P < 0.01) and it was higher than the data from the 1000 Genomes (36.60%) (31). In contrast, *PPARD* rs2016520 did not show a significant association with T2DM in our subjects, but the frequency of the C allele of rs2016520 (29.83%) was lower than that in the data from the 1000 Genomes (36.60%) (31). Comparison of our findings with the 1000 Genomes data showed that *PPARD* rs2016520 and rs3777744 showed dramatically different allele frequencies in different ethnic populations. The major reasons for this discrepancy may be the differences in specific ethnic groups and exposure to environmental factors. The main drivers of this variation may be due to differentiation in specific ethnic groups and the influence of exposure to diverse environments. The current study also displayed higher BMI and WHR values in T2DM patients with *PPARD* rs2016520 TC and CC genotypes, which indicated a potential contribution of the genetic variant to the prevalence of overweight and obesity in T2DM patients (**Table 2** and **Supplementary Figure S2**). In contrast, WHR, PPG, FINS and HOMA-IR were higher in patients with the rs3777744 risk G allele compared with those carrying the A allele (**Table 3** and **Supplementary Figure S3**). Potent associations have been determined between genetic variation in the *PPARD* gene and elevated susceptibility to T2DM, as well as obesity and insulin resistance. In addition, PPARδ is involved in regulating energy metabolism in liver, skeletal muscle and adipose tissue, and it is a mechanism by which *PPARD* gene variants lead to obesity and insulin resistance (32, 33).

The known involvement of PPARδ in insulin resistance, either directly or indirectly suggests that *PPARD* gene variants may account for individual differences in the clinical efficacy of exenatide. There is no evidence from previous studies that patients with specific *PPARD* gene variants have better or worse clinical efficacy of exenatide. In this study, we observed that the FINS and HOMA-IR levels of the subjects with at least one C allele of the *PPARD* rs2016520 were significantly lower than those with the TT genotype after 6 months of treatment, which suggested that the *PPARD* rs2016520 TT genotype conferred the poor exenatide response through a reduction of insulin resistance, as measured by HOMA-IR. PPARδ is engaged in glucose and lipid metabolism in the liver and exerts insulin-sensitizing effects, thereby improving hepatic insulin resistance (34). Therefore, our findings suggest that *PPARD* rs2016520 may affect the biological function of PPARδ, thereby influenceing the therapeutic effects of exenatide on improving insulin resistance of patients with T2DM.

The carriers of G alleles at rs3777744 exhibited higher levels of WHR, FPG, HbA1c and HOMA-IR compared to individuals with the AA genotype following 6 months of exenatide treatment, potentially accounting for the lower failure rate of exenatide therapy among the AA homozygotes. Additional analysis of metabolic features revealed that the G allele of rs3777744 had a detrimental effect on FPG and HOMA-IR, suggesting that an increased FPG and HbA1c may be caused, at minimum partially, by the negative effects of insulin resistance. Regarding rs3777744, which is located at intron2 of *PPARD* at chromosome 6p21.31, it is confirmed that the rs3777744 G allele is associated with cardiovascular disease risk in the Chinese population, though the underlying mechanism is unclear (35). A wealth of studies support the notion that cardiovascular disease is related to both insulin resistance and the compensatory hyperinsulinemia associated with insulin resistance (36).

Therefore, the genetic variants of *PPARD* might be responsible for interindividual differences in exenatide response. The underlying mechanisms leading to these findings remain unclear, but the effects of the *PPARD* rs3777744 G allele on IR, PPG and HbA1c need to be explored further to clarify the underlying mechanisms.

We hypothesize that the underlying mechanism by which *PPARD* variants contribute to individual differences in the development of T2DM and the therapeutic effects of exenatide is that PPARδ regulates the expression and function of GLP-1R. To fully illustrate the role of *PPARD* on exenatide efficacy and its associated pathways in IR, we established the insulin resistance model in HepG2 cells. Interestingly, evidence showed that SNPs in intron regions can affect gene function mainly by influencing splice site activity, while SNPs in the 5’-UTR may affect its binding to transcription factors, thus affecting protein expression and function (37). Therefore, we hypothesized that the regulation of *PPARD* gene expression in cells could resemble the genetic variation and functional changes of *PPARD* in the clinic. PPARδ is essentially a class of ligand-dependent transcriptional regulators, and GLP-1R is the pharmacological target of exenatide. We identified potential PPARδ binding sites by analyzing the GLP-1R promoter through the JASPAR database (**Supplementary Figure S1**). Therefore, we hypothesized that PPARδ regulates the level and function of GLP-1R and may be the underlying mechanism of *PPARD* gene variants. In this study, we found that the activation of PPARδ enhanced the uptake of extracellular glucose and the protein expression of GLP-1R in IR HepG2 cells, suggesting that PPARδ played a critical role in the regulation of insulin signaling pathways under pathological conditions. To further assess whether PPARδ is involved in the efficacy of exenatide, we examined the efficacy of exenatide on glucose uptake in the cellular level. In an IR cell model, we observed that activation of PPARδ potentiated the therapeutic benefits of exenatide in IR and the expression level of GLP-1R were significantly elevated. Consistent with the results of clinical trial, these data strongly suggest that exenatide increases insulin sensitivity in the liver, which could be further strengthened by overexpression of PPARδ. On the other hand, we knocked down PPARδ on HepG2 cells and performed the mentioned experiments and observed the opposite effect, corroborating with the above results.

Meanwhile, several shortcomings of this study need to be considered when interpreting our findings. First, the sample size is relatively small, which results in restricted statistical power and low frequency of mutant phenotypes, this may have led us to miss some meaningful results. The current study had an estimated 81-97% power (for α=0.05) to detect the difference in the parameters. Consequently, further detailed studies with expanded sample size are warranted to validate the effects of *PPARD* variants on exenatide efficacy. Second, individual differences are the product of interactions between multiple genetic and environmental factors. In the present study, different exenatide responses were found to be associated with *PPARD* gene variants, but it cannot be excluded that other susceptibility genetic variants are also involved in individual differences of exenatide therapeutic efficacy. Therefore, we will estimate the co-effect of multiple loci on the efficacy of exenatide in the following studies. Third, the different locations of *PPARD* rs2016520 (in the 5’-UTR) and rs3777744 (located in intron2) have made it more difficult to carry out functional studies. We only explored the regulatory effect of PPARδ on GLP-1R and its effect on exenatide efficacy, however, the mechanism of SNPs in *PPARD* influence on PPARδ has not been elucidated.

In conclusion, the *PPARD* variants appear to be associated with the therapeutic response to exenatide in patients with T2DM. There may be a link between PPARδ and GLP-1R in the diabetic condition, which may be the molecular mechanism by which *PPARD* gene variants influence T2DM risk, insulin resistance and clinical response to exenatide. Therefore, the *PPARD* risk mutations may serve as exenatide response predictors based on PPARδ regulating GLP-1R expression and mediating insulin resistance. More detailed pharmacogenetic and functional studies are needed to elucidate the exact effects of *PPARD* variants on exenatide therapeutic efficacy, which is necessary to lay the foundation for a more precise and patient-tailored therapy for T2DM.

## Data availability statement

The data presented in the study are deposited in the https://submit.ncbi.nlm.nih.gov/subs/biosample/SUB11888229/overview (NCBI) repository, accession number SUB11888229.

## Ethics statement

The studies involving human participants were reviewed and approved by Ethics Committee of the Affiliated Hospital of Xuzhou Medical University. The patients/participants provided their written informed consent to participate in this study. The animal study was reviewed and approved by Animal Experiment Ethics Review Committee of Xuzhou Medical University.

## Author contributions

JS contributed to conception of the article, data acquisition, statistical analysis, result interpretation, manuscript drafting and approved the final version. TW and XY designed the experiments and revised the paper. NL, RH, YY, KX, QL and TY helped with the experiments. HL contributed to data acquisition, and revised the paper. All authors contributed to the article and approved the submitted version.

## Funding

This work was supported by grants from the National Natural Science Foundation of China (82003866), the Science and Technology Planning Project of Xuzhou (KC20093), the Wuxi Science and Technology Development Medical and Health Guidance Project (No. CSZ0N1809), and Jiangsu Research Hospital Association for Precision Medication (No. JY202011).

## Acknowledgments

We thank all the volunteers in this study for their cooperation, and the physicians from the Department of Endocrinology, the Affiliated Hospital of Xuzhou Medical University for their support.

## Conflict of interest

The authors declare that the research was conducted in the absence of any commercial or financial relationships that could be construed as a potential conflict of interest.

## Publisher’s note

All claims expressed in this article are solely those of the authors and do not necessarily represent those of their affiliated organizations, or those of the publisher, the editors and the reviewers. Any product that may be evaluated in this article, or claim that may be made by its manufacturer, is not guaranteed or endorsed by the publisher.

## Supplementary material

Supplementary Figure 1 Predicted GLP-1R binding motif site sequence from the database JASPAR.

Supplementary Figure 2 Baseline levels of body mass index (BMI) **(A)**, waist to hip ratio (WHR) **(B)** and triglyceride (TG) **(C)** in T2DM patients with TT (n = 148), TC (n = 125) and CC (n = 27) genotypes of *PPARD* rs22016520. **P* < 0.05 and ***P*<0.01 compared with the TT genotype group, ^##^ *P*<0.01 compared with the TC genotype group (n = 300).

Supplementary Figure 3 Baseline levels of waist to hip ratio (WHR) **(A)**, postprandial plasma glucose (PPG) **(B)**, fasting serum insulin (FINS) **(C)** and homeostasis model assessment for insulin resistance (HOMA-IR) **(D)** in T2DM patients with AA (n = 95), AG (n = 150) or GG (n = 55) genotypes of *PPARD* rs3777744. **P* < 0.05, ***P* < 0.01 and ****P* < 0.001compared with the AA genotype group (n = 300).

Supplementary Figure 4 The expression of PPARδ decreased in IR HepG2 cell model and *db/db* mice. **(A)** The mRNA level of PPARδ in HepG2 cells was measured by RT-PCR. Data are expressed as the mean ± SE, n = 3. **(B)** The relative protein expression level of PPARδ in HepG2 cells was measured by Western blot. Data are expressed as the mean ± SE, n = 3. ^***^ *P* < 0.001 compared with NC. **(C)** The mRNA level of PPARδ in *db/db* mice was measured by RT-PCR. Data are expressed as the mean ± SE, n = 6. **(D)** The relative protein expression level of PPARδ in *db/db* mice was measured by Western blot. Data are expressed as the mean ± SE, n = 6. ^***^ *P* < 0.001 compared with *db/m*.

Supplementary Figure 5 Validation of PPARδ plasmid knockdown efficiency in HepG2 cells. **(A)** The mRNA level of PPARδ in HepG2 cells was measured by RT-PCR. **(B)** The relative protein expression level of PPARδ in HepG2 cells was measured by Western blot. Data are expressed as the mean ± SE, n = 3. ^*^ *P* < 0.05, ^**^ *P* < 0.01 and ^***^ *P* < 0.001 compared with siNC.
