# Supplementary material for: Effects of PPARD gene variants on the therapeutic responses to exenatide in chinese patients with type 2 diabetes mellitus
Source: Front Endocrinol (Lausanne). 2022 Aug 16;13:949990. doi: 10.3389/fendo.2022.949990 (PMC9424689; doi:10.3389/fendo.2022.949990)
Supplement: Supplementary file 1 [file DataSheet_1.docx]

Object IDs and corresponding URLs:

30183347: https://www.ncbi.nlm.nih.gov/biosample/30183347

30183348: https://www.ncbi.nlm.nih.gov/biosample/30183348

30183349: https://www.ncbi.nlm.nih.gov/biosample/30183349

30183350: https://www.ncbi.nlm.nih.gov/biosample/30183350

30183351: https://www.ncbi.nlm.nih.gov/biosample/30183351

30183352: https://www.ncbi.nlm.nih.gov/biosample/30183352

30183353: https://www.ncbi.nlm.nih.gov/biosample/30183353

30183354: https://www.ncbi.nlm.nih.gov/biosample/30183354

30183355: https://www.ncbi.nlm.nih.gov/biosample/30183355

30183356: https://www.ncbi.nlm.nih.gov/biosample/30183356

30183357: https://www.ncbi.nlm.nih.gov/biosample/30183357

30183358: https://www.ncbi.nlm.nih.gov/biosample/30183358

30183359: https://www.ncbi.nlm.nih.gov/biosample/30183359

30183360: https://www.ncbi.nlm.nih.gov/biosample/30183360

30183361: https://www.ncbi.nlm.nih.gov/biosample/30183361

30183362: https://www.ncbi.nlm.nih.gov/biosample/30183362

30183363: https://www.ncbi.nlm.nih.gov/biosample/30183363

30183364: https://www.ncbi.nlm.nih.gov/biosample/30183364

30183365: https://www.ncbi.nlm.nih.gov/biosample/30183365

30183366: https://www.ncbi.nlm.nih.gov/biosample/30183366

30183367: https://www.ncbi.nlm.nih.gov/biosample/30183367

30183368: https://www.ncbi.nlm.nih.gov/biosample/30183368

30183369: https://www.ncbi.nlm.nih.gov/biosample/30183369

30183370: https://www.ncbi.nlm.nih.gov/biosample/30183370

30183371: https://www.ncbi.nlm.nih.gov/biosample/30183371

30183372: https://www.ncbi.nlm.nih.gov/biosample/30183372

30183373: https://www.ncbi.nlm.nih.gov/biosample/30183373

30183374: https://www.ncbi.nlm.nih.gov/biosample/30183374

30183375: https://www.ncbi.nlm.nih.gov/biosample/30183375

30183376: https://www.ncbi.nlm.nih.gov/biosample/30183376

30183377: https://www.ncbi.nlm.nih.gov/biosample/30183377

30183378: https://www.ncbi.nlm.nih.gov/biosample/30183378

30183379: https://www.ncbi.nlm.nih.gov/biosample/30183379

30183380: https://www.ncbi.nlm.nih.gov/biosample/30183380

30183381: https://www.ncbi.nlm.nih.gov/biosample/30183381

30183382: https://www.ncbi.nlm.nih.gov/biosample/30183382

30183383: https://www.ncbi.nlm.nih.gov/biosample/30183383

30183384: https://www.ncbi.nlm.nih.gov/biosample/30183384

30183385: https://www.ncbi.nlm.nih.gov/biosample/30183385

30183386: https://www.ncbi.nlm.nih.gov/biosample/30183386

30183387: https://www.ncbi.nlm.nih.gov/biosample/30183387

30183388: https://www.ncbi.nlm.nih.gov/biosample/30183388

30183389: https://www.ncbi.nlm.nih.gov/biosample/30183389

30183390: https://www.ncbi.nlm.nih.gov/biosample/30183390

30183391: https://www.ncbi.nlm.nih.gov/biosample/30183391

30183392: https://www.ncbi.nlm.nih.gov/biosample/30183392

30183393: https://www.ncbi.nlm.nih.gov/biosample/30183393

30183394: https://www.ncbi.nlm.nih.gov/biosample/30183394

30183395: https://www.ncbi.nlm.nih.gov/biosample/30183395

30183396: https://www.ncbi.nlm.nih.gov/biosample/30183396

30183397: https://www.ncbi.nlm.nih.gov/biosample/30183397

30183398: https://www.ncbi.nlm.nih.gov/biosample/30183398

30183399: https://www.ncbi.nlm.nih.gov/biosample/30183399

30183400: https://www.ncbi.nlm.nih.gov/biosample/30183400

30183401: https://www.ncbi.nlm.nih.gov/biosample/30183401

30183402: https://www.ncbi.nlm.nih.gov/biosample/30183402

30183403: https://www.ncbi.nlm.nih.gov/biosample/30183403

30183404: https://www.ncbi.nlm.nih.gov/biosample/30183404

30183405: https://www.ncbi.nlm.nih.gov/biosample/30183405

30183406: https://www.ncbi.nlm.nih.gov/biosample/30183406

30183407: https://www.ncbi.nlm.nih.gov/biosample/30183407

30183408: https://www.ncbi.nlm.nih.gov/biosample/30183408

30183409: https://www.ncbi.nlm.nih.gov/biosample/30183409

30183410: https://www.ncbi.nlm.nih.gov/biosample/30183410

30183411: https://www.ncbi.nlm.nih.gov/biosample/30183411

30183412: https://www.ncbi.nlm.nih.gov/biosample/30183412

30183413: https://www.ncbi.nlm.nih.gov/biosample/30183413

30183414: https://www.ncbi.nlm.nih.gov/biosample/30183414

30183415: https://www.ncbi.nlm.nih.gov/biosample/30183415

30183416: https://www.ncbi.nlm.nih.gov/biosample/30183416

30183417: https://www.ncbi.nlm.nih.gov/biosample/30183417

30183418: https://www.ncbi.nlm.nih.gov/biosample/30183418

30183419: https://www.ncbi.nlm.nih.gov/biosample/30183419

30183420: https://www.ncbi.nlm.nih.gov/biosample/30183420

30183421: https://www.ncbi.nlm.nih.gov/biosample/30183421

30183422: https://www.ncbi.nlm.nih.gov/biosample/30183422

30183423: https://www.ncbi.nlm.nih.gov/biosample/30183423

30183424: https://www.ncbi.nlm.nih.gov/biosample/30183424

30183425: https://www.ncbi.nlm.nih.gov/biosample/30183425

30183426: https://www.ncbi.nlm.nih.gov/biosample/30183426

30183427: https://www.ncbi.nlm.nih.gov/biosample/30183427

30183428: https://www.ncbi.nlm.nih.gov/biosample/30183428

30183429: https://www.ncbi.nlm.nih.gov/biosample/30183429

30183430: https://www.ncbi.nlm.nih.gov/biosample/30183430

30183431: https://www.ncbi.nlm.nih.gov/biosample/30183431

30183432: https://www.ncbi.nlm.nih.gov/biosample/30183432

30183433: https://www.ncbi.nlm.nih.gov/biosample/30183433

30183434: https://www.ncbi.nlm.nih.gov/biosample/30183434

30183435: https://www.ncbi.nlm.nih.gov/biosample/30183435

30183436: https://www.ncbi.nlm.nih.gov/biosample/30183436

30183437: https://www.ncbi.nlm.nih.gov/biosample/30183437

30183438: https://www.ncbi.nlm.nih.gov/biosample/30183438

30183439: https://www.ncbi.nlm.nih.gov/biosample/30183439

30183440: https://www.ncbi.nlm.nih.gov/biosample/30183440

30183441: https://www.ncbi.nlm.nih.gov/biosample/30183441

30183442: https://www.ncbi.nlm.nih.gov/biosample/30183442

30183443: https://www.ncbi.nlm.nih.gov/biosample/30183443

30183444: https://www.ncbi.nlm.nih.gov/biosample/30183444

30183445: https://www.ncbi.nlm.nih.gov/biosample/30183445

30183446: https://www.ncbi.nlm.nih.gov/biosample/30183446

30183447: https://www.ncbi.nlm.nih.gov/biosample/30183447

30183448: https://www.ncbi.nlm.nih.gov/biosample/30183448

30183449: https://www.ncbi.nlm.nih.gov/biosample/30183449

30183450: https://www.ncbi.nlm.nih.gov/biosample/30183450

30183451: https://www.ncbi.nlm.nih.gov/biosample/30183451

30183452: https://www.ncbi.nlm.nih.gov/biosample/30183452

30183453: https://www.ncbi.nlm.nih.gov/biosample/30183453

30183454: https://www.ncbi.nlm.nih.gov/biosample/30183454

30183455: https://www.ncbi.nlm.nih.gov/biosample/30183455

30183456: https://www.ncbi.nlm.nih.gov/biosample/30183456

30183457: https://www.ncbi.nlm.nih.gov/biosample/30183457

30183458: https://www.ncbi.nlm.nih.gov/biosample/30183458

30183459: https://www.ncbi.nlm.nih.gov/biosample/30183459

30183460: https://www.ncbi.nlm.nih.gov/biosample/30183460

30183461: https://www.ncbi.nlm.nih.gov/biosample/30183461

30183462: https://www.ncbi.nlm.nih.gov/biosample/30183462

30183463: https://www.ncbi.nlm.nih.gov/biosample/30183463

30183464: https://www.ncbi.nlm.nih.gov/biosample/30183464

30183465: https://www.ncbi.nlm.nih.gov/biosample/30183465

30183466: https://www.ncbi.nlm.nih.gov/biosample/30183466

30183467: https://www.ncbi.nlm.nih.gov/biosample/30183467

30183468: https://www.ncbi.nlm.nih.gov/biosample/30183468

30183469: https://www.ncbi.nlm.nih.gov/biosample/30183469

30183470: https://www.ncbi.nlm.nih.gov/biosample/30183470

30183471: https://www.ncbi.nlm.nih.gov/biosample/30183471

30183472: https://www.ncbi.nlm.nih.gov/biosample/30183472

30183473: https://www.ncbi.nlm.nih.gov/biosample/30183473

30183474: https://www.ncbi.nlm.nih.gov/biosample/30183474

30183475: https://www.ncbi.nlm.nih.gov/biosample/30183475

30183476: https://www.ncbi.nlm.nih.gov/biosample/30183476

30183477: https://www.ncbi.nlm.nih.gov/biosample/30183477

30183478: https://www.ncbi.nlm.nih.gov/biosample/30183478

30183479: https://www.ncbi.nlm.nih.gov/biosample/30183479

30183480: https://www.ncbi.nlm.nih.gov/biosample/30183480

30183481: https://www.ncbi.nlm.nih.gov/biosample/30183481

30183482: https://www.ncbi.nlm.nih.gov/biosample/30183482

30183483: https://www.ncbi.nlm.nih.gov/biosample/30183483

30183484: https://www.ncbi.nlm.nih.gov/biosample/30183484

30183485: https://www.ncbi.nlm.nih.gov/biosample/30183485

30183486: https://www.ncbi.nlm.nih.gov/biosample/30183486

30183487: https://www.ncbi.nlm.nih.gov/biosample/30183487

30183488: https://www.ncbi.nlm.nih.gov/biosample/30183488

30183489: https://www.ncbi.nlm.nih.gov/biosample/30183489

30183490: https://www.ncbi.nlm.nih.gov/biosample/30183490

30183491: https://www.ncbi.nlm.nih.gov/biosample/30183491

30183492: https://www.ncbi.nlm.nih.gov/biosample/30183492

30183493: https://www.ncbi.nlm.nih.gov/biosample/30183493

30183494: https://www.ncbi.nlm.nih.gov/biosample/30183494

30183495: https://www.ncbi.nlm.nih.gov/biosample/30183495

30183496: https://www.ncbi.nlm.nih.gov/biosample/30183496

30183497: https://www.ncbi.nlm.nih.gov/biosample/30183497

30183498: https://www.ncbi.nlm.nih.gov/biosample/30183498

30183499: https://www.ncbi.nlm.nih.gov/biosample/30183499

30183500: https://www.ncbi.nlm.nih.gov/biosample/30183500

30183501: https://www.ncbi.nlm.nih.gov/biosample/30183501

30183502: https://www.ncbi.nlm.nih.gov/biosample/30183502

30183503: https://www.ncbi.nlm.nih.gov/biosample/30183503

30183504: https://www.ncbi.nlm.nih.gov/biosample/30183504

30183505: https://www.ncbi.nlm.nih.gov/biosample/30183505

30183506: https://www.ncbi.nlm.nih.gov/biosample/30183506

30183507: https://www.ncbi.nlm.nih.gov/biosample/30183507

30183508: https://www.ncbi.nlm.nih.gov/biosample/30183508

30183509: https://www.ncbi.nlm.nih.gov/biosample/30183509

30183510: https://www.ncbi.nlm.nih.gov/biosample/30183510

30183511: https://www.ncbi.nlm.nih.gov/biosample/30183511

30183512: https://www.ncbi.nlm.nih.gov/biosample/30183512

30183513: https://www.ncbi.nlm.nih.gov/biosample/30183513

30183514: https://www.ncbi.nlm.nih.gov/biosample/30183514

30183515: https://www.ncbi.nlm.nih.gov/biosample/30183515

30183516: https://www.ncbi.nlm.nih.gov/biosample/30183516

30183517: https://www.ncbi.nlm.nih.gov/biosample/30183517

30183518: https://www.ncbi.nlm.nih.gov/biosample/30183518

30183519: https://www.ncbi.nlm.nih.gov/biosample/30183519

30183520: https://www.ncbi.nlm.nih.gov/biosample/30183520

30183521: https://www.ncbi.nlm.nih.gov/biosample/30183521

30183522: https://www.ncbi.nlm.nih.gov/biosample/30183522

30183523: https://www.ncbi.nlm.nih.gov/biosample/30183523

30183524: https://www.ncbi.nlm.nih.gov/biosample/30183524

30183525: https://www.ncbi.nlm.nih.gov/biosample/30183525

30183526: https://www.ncbi.nlm.nih.gov/biosample/30183526

30183527: https://www.ncbi.nlm.nih.gov/biosample/30183527

30183528: https://www.ncbi.nlm.nih.gov/biosample/30183528

30183529: https://www.ncbi.nlm.nih.gov/biosample/30183529

30183530: https://www.ncbi.nlm.nih.gov/biosample/30183530

30183531: https://www.ncbi.nlm.nih.gov/biosample/30183531

30183532: https://www.ncbi.nlm.nih.gov/biosample/30183532

30183533: https://www.ncbi.nlm.nih.gov/biosample/30183533

30183534: https://www.ncbi.nlm.nih.gov/biosample/30183534

30183535: https://www.ncbi.nlm.nih.gov/biosample/30183535

30183536: https://www.ncbi.nlm.nih.gov/biosample/30183536

30183537: https://www.ncbi.nlm.nih.gov/biosample/30183537

30183538: https://www.ncbi.nlm.nih.gov/biosample/30183538

30183539: https://www.ncbi.nlm.nih.gov/biosample/30183539

30183540: https://www.ncbi.nlm.nih.gov/biosample/30183540

30183541: https://www.ncbi.nlm.nih.gov/biosample/30183541

30183542: https://www.ncbi.nlm.nih.gov/biosample/30183542

30183543: https://www.ncbi.nlm.nih.gov/biosample/30183543

30183544: https://www.ncbi.nlm.nih.gov/biosample/30183544

30183545: https://www.ncbi.nlm.nih.gov/biosample/30183545

30183546: https://www.ncbi.nlm.nih.gov/biosample/30183546

30183547: https://www.ncbi.nlm.nih.gov/biosample/30183547

30183548: https://www.ncbi.nlm.nih.gov/biosample/30183548

30183549: https://www.ncbi.nlm.nih.gov/biosample/30183549

30183550: https://www.ncbi.nlm.nih.gov/biosample/30183550

30183551: https://www.ncbi.nlm.nih.gov/biosample/30183551

30183552: https://www.ncbi.nlm.nih.gov/biosample/30183552

30183553: https://www.ncbi.nlm.nih.gov/biosample/30183553

30183554: https://www.ncbi.nlm.nih.gov/biosample/30183554

30183555: https://www.ncbi.nlm.nih.gov/biosample/30183555

30183556: https://www.ncbi.nlm.nih.gov/biosample/30183556

30183557: https://www.ncbi.nlm.nih.gov/biosample/30183557

30183558: https://www.ncbi.nlm.nih.gov/biosample/30183558

30183559: https://www.ncbi.nlm.nih.gov/biosample/30183559

30183560: https://www.ncbi.nlm.nih.gov/biosample/30183560

30183561: https://www.ncbi.nlm.nih.gov/biosample/30183561

30183562: https://www.ncbi.nlm.nih.gov/biosample/30183562

30183563: https://www.ncbi.nlm.nih.gov/biosample/30183563

30183564: https://www.ncbi.nlm.nih.gov/biosample/30183564

30183565: https://www.ncbi.nlm.nih.gov/biosample/30183565

30183566: https://www.ncbi.nlm.nih.gov/biosample/30183566

30183567: https://www.ncbi.nlm.nih.gov/biosample/30183567

30183568: https://www.ncbi.nlm.nih.gov/biosample/30183568

30183569: https://www.ncbi.nlm.nih.gov/biosample/30183569

30183570: https://www.ncbi.nlm.nih.gov/biosample/30183570

30183571: https://www.ncbi.nlm.nih.gov/biosample/30183571

30183572: https://www.ncbi.nlm.nih.gov/biosample/30183572

30183573: https://www.ncbi.nlm.nih.gov/biosample/30183573

30183574: https://www.ncbi.nlm.nih.gov/biosample/30183574

30183575: https://www.ncbi.nlm.nih.gov/biosample/30183575

30183576: https://www.ncbi.nlm.nih.gov/biosample/30183576

30183577: https://www.ncbi.nlm.nih.gov/biosample/30183577

30183578: https://www.ncbi.nlm.nih.gov/biosample/30183578

30183579: https://www.ncbi.nlm.nih.gov/biosample/30183579

30183580: https://www.ncbi.nlm.nih.gov/biosample/30183580

30183581: https://www.ncbi.nlm.nih.gov/biosample/30183581

30183582: https://www.ncbi.nlm.nih.gov/biosample/30183582

30183583: https://www.ncbi.nlm.nih.gov/biosample/30183583

30183584: https://www.ncbi.nlm.nih.gov/biosample/30183584

30183585: https://www.ncbi.nlm.nih.gov/biosample/30183585

30183586: https://www.ncbi.nlm.nih.gov/biosample/30183586

30183587: https://www.ncbi.nlm.nih.gov/biosample/30183587

30183588: https://www.ncbi.nlm.nih.gov/biosample/30183588

30183589: https://www.ncbi.nlm.nih.gov/biosample/30183589

30183590: https://www.ncbi.nlm.nih.gov/biosample/30183590

30183591: https://www.ncbi.nlm.nih.gov/biosample/30183591

30183592: https://www.ncbi.nlm.nih.gov/biosample/30183592

30183593: https://www.ncbi.nlm.nih.gov/biosample/30183593

30183594: https://www.ncbi.nlm.nih.gov/biosample/30183594

30183595: https://www.ncbi.nlm.nih.gov/biosample/30183595

30183596: https://www.ncbi.nlm.nih.gov/biosample/30183596

30183597: https://www.ncbi.nlm.nih.gov/biosample/30183597

30183598: https://www.ncbi.nlm.nih.gov/biosample/30183598

30183599: https://www.ncbi.nlm.nih.gov/biosample/30183599

30183600: https://www.ncbi.nlm.nih.gov/biosample/30183600

30183601: https://www.ncbi.nlm.nih.gov/biosample/30183601

30183602: https://www.ncbi.nlm.nih.gov/biosample/30183602

30183603: https://www.ncbi.nlm.nih.gov/biosample/30183603

30183604: https://www.ncbi.nlm.nih.gov/biosample/30183604

30183605: https://www.ncbi.nlm.nih.gov/biosample/30183605

30183606: https://www.ncbi.nlm.nih.gov/biosample/30183606

30183607: https://www.ncbi.nlm.nih.gov/biosample/30183607

30183608: https://www.ncbi.nlm.nih.gov/biosample/30183608

30183609: https://www.ncbi.nlm.nih.gov/biosample/30183609

30183610: https://www.ncbi.nlm.nih.gov/biosample/30183610

30183611: https://www.ncbi.nlm.nih.gov/biosample/30183611

30183612: https://www.ncbi.nlm.nih.gov/biosample/30183612

30183613: https://www.ncbi.nlm.nih.gov/biosample/30183613

30183614: https://www.ncbi.nlm.nih.gov/biosample/30183614

30183615: https://www.ncbi.nlm.nih.gov/biosample/30183615

30183616: https://www.ncbi.nlm.nih.gov/biosample/30183616

30183617: https://www.ncbi.nlm.nih.gov/biosample/30183617

30183618: https://www.ncbi.nlm.nih.gov/biosample/30183618

30183619: https://www.ncbi.nlm.nih.gov/biosample/30183619

30183620: https://www.ncbi.nlm.nih.gov/biosample/30183620

30183621: https://www.ncbi.nlm.nih.gov/biosample/30183621

30183622: https://www.ncbi.nlm.nih.gov/biosample/30183622

30183623: https://www.ncbi.nlm.nih.gov/biosample/30183623

30183624: https://www.ncbi.nlm.nih.gov/biosample/30183624

30183625: https://www.ncbi.nlm.nih.gov/biosample/30183625

30183626: https://www.ncbi.nlm.nih.gov/biosample/30183626

30183627: https://www.ncbi.nlm.nih.gov/biosample/30183627

30183628: https://www.ncbi.nlm.nih.gov/biosample/30183628

30183629: https://www.ncbi.nlm.nih.gov/biosample/30183629

30183630: https://www.ncbi.nlm.nih.gov/biosample/30183630

30183631: https://www.ncbi.nlm.nih.gov/biosample/30183631

30183632: https://www.ncbi.nlm.nih.gov/biosample/30183632

30183633: https://www.ncbi.nlm.nih.gov/biosample/30183633

30183634: https://www.ncbi.nlm.nih.gov/biosample/30183634

30183635: https://www.ncbi.nlm.nih.gov/biosample/30183635

30183636: https://www.ncbi.nlm.nih.gov/biosample/30183636

30183637: https://www.ncbi.nlm.nih.gov/biosample/30183637

30183638: https://www.ncbi.nlm.nih.gov/biosample/30183638

30183639: https://www.ncbi.nlm.nih.gov/biosample/30183639

30183640: https://www.ncbi.nlm.nih.gov/biosample/30183640

30183641: https://www.ncbi.nlm.nih.gov/biosample/30183641

30183642: https://www.ncbi.nlm.nih.gov/biosample/30183642

30183643: https://www.ncbi.nlm.nih.gov/biosample/30183643

30183644: https://www.ncbi.nlm.nih.gov/biosample/30183644

30183645: https://www.ncbi.nlm.nih.gov/biosample/30183645

30183646: https://www.ncbi.nlm.nih.gov/biosample/30183646

30183647: https://www.ncbi.nlm.nih.gov/biosample/30183647

30183648: https://www.ncbi.nlm.nih.gov/biosample/30183648

30183649: https://www.ncbi.nlm.nih.gov/biosample/30183649

30183650: https://www.ncbi.nlm.nih.gov/biosample/30183650

30183651: https://www.ncbi.nlm.nih.gov/biosample/30183651

30183652: https://www.ncbi.nlm.nih.gov/biosample/30183652

30183653: https://www.ncbi.nlm.nih.gov/biosample/30183653

30183654: https://www.ncbi.nlm.nih.gov/biosample/30183654

30183655: https://www.ncbi.nlm.nih.gov/biosample/30183655

30183656: https://www.ncbi.nlm.nih.gov/biosample/30183656

30183657: https://www.ncbi.nlm.nih.gov/biosample/30183657

30183658: https://www.ncbi.nlm.nih.gov/biosample/30183658

30183659: https://www.ncbi.nlm.nih.gov/biosample/30183659

30183660: https://www.ncbi.nlm.nih.gov/biosample/30183660

30183661: https://www.ncbi.nlm.nih.gov/biosample/30183661

30183662: https://www.ncbi.nlm.nih.gov/biosample/30183662

30183663: https://www.ncbi.nlm.nih.gov/biosample/30183663

30183664: https://www.ncbi.nlm.nih.gov/biosample/30183664

30183665: https://www.ncbi.nlm.nih.gov/biosample/30183665

30183666: https://www.ncbi.nlm.nih.gov/biosample/30183666

30183667: https://www.ncbi.nlm.nih.gov/biosample/30183667

30183668: https://www.ncbi.nlm.nih.gov/biosample/30183668

30183669: https://www.ncbi.nlm.nih.gov/biosample/30183669

30183670: https://www.ncbi.nlm.nih.gov/biosample/30183670

30183671: https://www.ncbi.nlm.nih.gov/biosample/30183671

30183672: https://www.ncbi.nlm.nih.gov/biosample/30183672

30183673: https://www.ncbi.nlm.nih.gov/biosample/30183673

30183674: https://www.ncbi.nlm.nih.gov/biosample/30183674

30183675: https://www.ncbi.nlm.nih.gov/biosample/30183675

30183676: https://www.ncbi.nlm.nih.gov/biosample/30183676

30183677: https://www.ncbi.nlm.nih.gov/biosample/30183677

30183678: https://www.ncbi.nlm.nih.gov/biosample/30183678

30183679: https://www.ncbi.nlm.nih.gov/biosample/30183679

30183680: https://www.ncbi.nlm.nih.gov/biosample/30183680

30183681: https://www.ncbi.nlm.nih.gov/biosample/30183681

30183682: https://www.ncbi.nlm.nih.gov/biosample/30183682

30183683: https://www.ncbi.nlm.nih.gov/biosample/30183683

30183684: https://www.ncbi.nlm.nih.gov/biosample/30183684

30183685: https://www.ncbi.nlm.nih.gov/biosample/30183685

30183686: https://www.ncbi.nlm.nih.gov/biosample/30183686

30183687: https://www.ncbi.nlm.nih.gov/biosample/30183687

30183688: https://www.ncbi.nlm.nih.gov/biosample/30183688

30183689: https://www.ncbi.nlm.nih.gov/biosample/30183689

30183690: https://www.ncbi.nlm.nih.gov/biosample/30183690

30183691: https://www.ncbi.nlm.nih.gov/biosample/30183691

30183692: https://www.ncbi.nlm.nih.gov/biosample/30183692

30183693: https://www.ncbi.nlm.nih.gov/biosample/30183693

30183694: https://www.ncbi.nlm.nih.gov/biosample/30183694

30183695: https://www.ncbi.nlm.nih.gov/biosample/30183695

30183696: https://www.ncbi.nlm.nih.gov/biosample/30183696

30183697: https://www.ncbi.nlm.nih.gov/biosample/30183697

30183698: https://www.ncbi.nlm.nih.gov/biosample/30183698

30183699: https://www.ncbi.nlm.nih.gov/biosample/30183699

30183700: https://www.ncbi.nlm.nih.gov/biosample/30183700

30183701: https://www.ncbi.nlm.nih.gov/biosample/30183701

30183702: https://www.ncbi.nlm.nih.gov/biosample/30183702

30183703: https://www.ncbi.nlm.nih.gov/biosample/30183703

30183704: https://www.ncbi.nlm.nih.gov/biosample/30183704

30183705: https://www.ncbi.nlm.nih.gov/biosample/30183705

30183706: https://www.ncbi.nlm.nih.gov/biosample/30183706

30183707: https://www.ncbi.nlm.nih.gov/biosample/30183707

30183708: https://www.ncbi.nlm.nih.gov/biosample/30183708

30183709: https://www.ncbi.nlm.nih.gov/biosample/30183709

30183710: https://www.ncbi.nlm.nih.gov/biosample/30183710

30183711: https://www.ncbi.nlm.nih.gov/biosample/30183711

30183712: https://www.ncbi.nlm.nih.gov/biosample/30183712

30183713: https://www.ncbi.nlm.nih.gov/biosample/30183713

30183714: https://www.ncbi.nlm.nih.gov/biosample/30183714

30183715: https://www.ncbi.nlm.nih.gov/biosample/30183715

30183716: https://www.ncbi.nlm.nih.gov/biosample/30183716

30183717: https://www.ncbi.nlm.nih.gov/biosample/30183717

30183718: https://www.ncbi.nlm.nih.gov/biosample/30183718

30183719: https://www.ncbi.nlm.nih.gov/biosample/30183719

30183720: https://www.ncbi.nlm.nih.gov/biosample/30183720

30183721: https://www.ncbi.nlm.nih.gov/biosample/30183721

30183722: https://www.ncbi.nlm.nih.gov/biosample/30183722

30183723: https://www.ncbi.nlm.nih.gov/biosample/30183723

30183724: https://www.ncbi.nlm.nih.gov/biosample/30183724

30183725: https://www.ncbi.nlm.nih.gov/biosample/30183725

30183726: https://www.ncbi.nlm.nih.gov/biosample/30183726

30183727: https://www.ncbi.nlm.nih.gov/biosample/30183727

30183728: https://www.ncbi.nlm.nih.gov/biosample/30183728

30183729: https://www.ncbi.nlm.nih.gov/biosample/30183729

30183730: https://www.ncbi.nlm.nih.gov/biosample/30183730

30183731: https://www.ncbi.nlm.nih.gov/biosample/30183731

30183732: https://www.ncbi.nlm.nih.gov/biosample/30183732

30183733: https://www.ncbi.nlm.nih.gov/biosample/30183733

30183734: https://www.ncbi.nlm.nih.gov/biosample/30183734

30183735: https://www.ncbi.nlm.nih.gov/biosample/30183735

30183736: https://www.ncbi.nlm.nih.gov/biosample/30183736

30183737: https://www.ncbi.nlm.nih.gov/biosample/30183737

30183738: https://www.ncbi.nlm.nih.gov/biosample/30183738

30183739: https://www.ncbi.nlm.nih.gov/biosample/30183739

30183740: https://www.ncbi.nlm.nih.gov/biosample/30183740

30183741: https://www.ncbi.nlm.nih.gov/biosample/30183741

30183742: https://www.ncbi.nlm.nih.gov/biosample/30183742

30183743: https://www.ncbi.nlm.nih.gov/biosample/30183743

30183744: https://www.ncbi.nlm.nih.gov/biosample/30183744

30183745: https://www.ncbi.nlm.nih.gov/biosample/30183745

30183746: https://www.ncbi.nlm.nih.gov/biosample/30183746

30183747: https://www.ncbi.nlm.nih.gov/biosample/30183747

30183748: https://www.ncbi.nlm.nih.gov/biosample/30183748

30183749: https://www.ncbi.nlm.nih.gov/biosample/30183749

30183750: https://www.ncbi.nlm.nih.gov/biosample/30183750

30183751: https://www.ncbi.nlm.nih.gov/biosample/30183751

30183752: https://www.ncbi.nlm.nih.gov/biosample/30183752

30183753: https://www.ncbi.nlm.nih.gov/biosample/30183753

30183754: https://www.ncbi.nlm.nih.gov/biosample/30183754

30183755: https://www.ncbi.nlm.nih.gov/biosample/30183755

30183756: https://www.ncbi.nlm.nih.gov/biosample/30183756

30183757: https://www.ncbi.nlm.nih.gov/biosample/30183757

30183758: https://www.ncbi.nlm.nih.gov/biosample/30183758

30183759: https://www.ncbi.nlm.nih.gov/biosample/30183759

30183760: https://www.ncbi.nlm.nih.gov/biosample/30183760

30183761: https://www.ncbi.nlm.nih.gov/biosample/30183761

30183762: https://www.ncbi.nlm.nih.gov/biosample/30183762

30183763: https://www.ncbi.nlm.nih.gov/biosample/30183763

30183764: https://www.ncbi.nlm.nih.gov/biosample/30183764

30183765: https://www.ncbi.nlm.nih.gov/biosample/30183765

30183766: https://www.ncbi.nlm.nih.gov/biosample/30183766

30183767: https://www.ncbi.nlm.nih.gov/biosample/30183767

30183768: https://www.ncbi.nlm.nih.gov/biosample/30183768

30183769: https://www.ncbi.nlm.nih.gov/biosample/30183769

30183770: https://www.ncbi.nlm.nih.gov/biosample/30183770

30183771: https://www.ncbi.nlm.nih.gov/biosample/30183771

30183772: https://www.ncbi.nlm.nih.gov/biosample/30183772

30183773: https://www.ncbi.nlm.nih.gov/biosample/30183773

30183774: https://www.ncbi.nlm.nih.gov/biosample/30183774

30183775: https://www.ncbi.nlm.nih.gov/biosample/30183775

30183776: https://www.ncbi.nlm.nih.gov/biosample/30183776

30183777: https://www.ncbi.nlm.nih.gov/biosample/30183777

30183778: https://www.ncbi.nlm.nih.gov/biosample/30183778

30183779: https://www.ncbi.nlm.nih.gov/biosample/30183779

30183780: https://www.ncbi.nlm.nih.gov/biosample/30183780

30183781: https://www.ncbi.nlm.nih.gov/biosample/30183781

30183782: https://www.ncbi.nlm.nih.gov/biosample/30183782

30183783: https://www.ncbi.nlm.nih.gov/biosample/30183783

30183784: https://www.ncbi.nlm.nih.gov/biosample/30183784

30183785: https://www.ncbi.nlm.nih.gov/biosample/30183785

30183786: https://www.ncbi.nlm.nih.gov/biosample/30183786

30183787: https://www.ncbi.nlm.nih.gov/biosample/30183787

30183788: https://www.ncbi.nlm.nih.gov/biosample/30183788

30183789: https://www.ncbi.nlm.nih.gov/biosample/30183789

30183790: https://www.ncbi.nlm.nih.gov/biosample/30183790

30183791: https://www.ncbi.nlm.nih.gov/biosample/30183791

30183792: https://www.ncbi.nlm.nih.gov/biosample/30183792

30183793: https://www.ncbi.nlm.nih.gov/biosample/30183793

30183794: https://www.ncbi.nlm.nih.gov/biosample/30183794

30183795: https://www.ncbi.nlm.nih.gov/biosample/30183795

30183796: https://www.ncbi.nlm.nih.gov/biosample/30183796

30183797: https://www.ncbi.nlm.nih.gov/biosample/30183797

30183798: https://www.ncbi.nlm.nih.gov/biosample/30183798

30183799: https://www.ncbi.nlm.nih.gov/biosample/30183799

30183800: https://www.ncbi.nlm.nih.gov/biosample/30183800

30183801: https://www.ncbi.nlm.nih.gov/biosample/30183801

30183802: https://www.ncbi.nlm.nih.gov/biosample/30183802

30183803: https://www.ncbi.nlm.nih.gov/biosample/30183803

30183804: https://www.ncbi.nlm.nih.gov/biosample/30183804

30183805: https://www.ncbi.nlm.nih.gov/biosample/30183805

30183806: https://www.ncbi.nlm.nih.gov/biosample/30183806

30183807: https://www.ncbi.nlm.nih.gov/biosample/30183807

30183808: https://www.ncbi.nlm.nih.gov/biosample/30183808

30183809: https://www.ncbi.nlm.nih.gov/biosample/30183809

30183810: https://www.ncbi.nlm.nih.gov/biosample/30183810

30183811: https://www.ncbi.nlm.nih.gov/biosample/30183811

30183812: https://www.ncbi.nlm.nih.gov/biosample/30183812

30183813: https://www.ncbi.nlm.nih.gov/biosample/30183813

30183814: https://www.ncbi.nlm.nih.gov/biosample/30183814

30183815: https://www.ncbi.nlm.nih.gov/biosample/30183815

30183816: https://www.ncbi.nlm.nih.gov/biosample/30183816

30183817: https://www.ncbi.nlm.nih.gov/biosample/30183817

30183818: https://www.ncbi.nlm.nih.gov/biosample/30183818

30183819: https://www.ncbi.nlm.nih.gov/biosample/30183819

30183820: https://www.ncbi.nlm.nih.gov/biosample/30183820

30183821: https://www.ncbi.nlm.nih.gov/biosample/30183821

30183822: https://www.ncbi.nlm.nih.gov/biosample/30183822

30183823: https://www.ncbi.nlm.nih.gov/biosample/30183823

30183824: https://www.ncbi.nlm.nih.gov/biosample/30183824

30183825: https://www.ncbi.nlm.nih.gov/biosample/30183825

30183826: https://www.ncbi.nlm.nih.gov/biosample/30183826

30183827: https://www.ncbi.nlm.nih.gov/biosample/30183827

30183828: https://www.ncbi.nlm.nih.gov/biosample/30183828

30183829: https://www.ncbi.nlm.nih.gov/biosample/30183829

30183830: https://www.ncbi.nlm.nih.gov/biosample/30183830

30183831: https://www.ncbi.nlm.nih.gov/biosample/30183831

30183832: https://www.ncbi.nlm.nih.gov/biosample/30183832

30183833: https://www.ncbi.nlm.nih.gov/biosample/30183833

30183834: https://www.ncbi.nlm.nih.gov/biosample/30183834

30183835: https://www.ncbi.nlm.nih.gov/biosample/30183835

30183836: https://www.ncbi.nlm.nih.gov/biosample/30183836

30183837: https://www.ncbi.nlm.nih.gov/biosample/30183837

30183838: https://www.ncbi.nlm.nih.gov/biosample/30183838

30183839: https://www.ncbi.nlm.nih.gov/biosample/30183839

30183840: https://www.ncbi.nlm.nih.gov/biosample/30183840

30183841: https://www.ncbi.nlm.nih.gov/biosample/30183841

30183842: https://www.ncbi.nlm.nih.gov/biosample/30183842

30183843: https://www.ncbi.nlm.nih.gov/biosample/30183843

30183844: https://www.ncbi.nlm.nih.gov/biosample/30183844

30183845: https://www.ncbi.nlm.nih.gov/biosample/30183845

30183846: <https://www.ncbi.nlm.nih.gov/biosample/30183846>

Accession Sample Name SPUID Organism Tax ID Isolate

SAMN30183347 T2DM-0001 T2DM-0001 Homo sapiens 9606 blood

SAMN30183348 T2DM-0002 T2DM-0002 Homo sapiens 9606 blood

SAMN30183349 T2DM-0003 T2DM-0003 Homo sapiens 9606 blood

SAMN30183350 T2DM-0004 T2DM-0004 Homo sapiens 9606 blood

SAMN30183351 T2DM-0005 T2DM-0005 Homo sapiens 9606 blood

SAMN30183352 T2DM-0006 T2DM-0006 Homo sapiens 9606 blood

SAMN30183353 T2DM-0007 T2DM-0007 Homo sapiens 9606 blood

SAMN30183354 T2DM-0008 T2DM-0008 Homo sapiens 9606 blood

SAMN30183355 T2DM-0009 T2DM-0009 Homo sapiens 9606 blood

SAMN30183356 T2DM-0010 T2DM-0010 Homo sapiens 9606 blood

SAMN30183357 T2DM-0011 T2DM-0011 Homo sapiens 9606 blood

SAMN30183358 T2DM-0012 T2DM-0012 Homo sapiens 9606 blood

SAMN30183359 T2DM-0013 T2DM-0013 Homo sapiens 9606 blood

SAMN30183360 T2DM-0014 T2DM-0014 Homo sapiens 9606 blood

SAMN30183361 T2DM-0015 T2DM-0015 Homo sapiens 9606 blood

SAMN30183362 T2DM-0016 T2DM-0016 Homo sapiens 9606 blood

SAMN30183363 T2DM-0017 T2DM-0017 Homo sapiens 9606 blood

SAMN30183364 T2DM-0018 T2DM-0018 Homo sapiens 9606 blood

SAMN30183365 T2DM-0019 T2DM-0019 Homo sapiens 9606 blood

SAMN30183366 T2DM-0020 T2DM-0020 Homo sapiens 9606 blood

SAMN30183367 T2DM-0021 T2DM-0021 Homo sapiens 9606 blood

SAMN30183368 T2DM-0022 T2DM-0022 Homo sapiens 9606 blood

SAMN30183369 T2DM-0023 T2DM-0023 Homo sapiens 9606 blood

SAMN30183370 T2DM-0024 T2DM-0024 Homo sapiens 9606 blood

SAMN30183371 T2DM-0025 T2DM-0025 Homo sapiens 9606 blood

SAMN30183372 T2DM-0026 T2DM-0026 Homo sapiens 9606 blood

SAMN30183373 T2DM-0027 T2DM-0027 Homo sapiens 9606 blood

SAMN30183374 T2DM-0028 T2DM-0028 Homo sapiens 9606 blood

SAMN30183375 T2DM-0029 T2DM-0029 Homo sapiens 9606 blood

SAMN30183376 T2DM-0030 T2DM-0030 Homo sapiens 9606 blood

SAMN30183377 T2DM-0031 T2DM-0031 Homo sapiens 9606 blood

SAMN30183378 T2DM-0032 T2DM-0032 Homo sapiens 9606 blood

SAMN30183379 T2DM-0033 T2DM-0033 Homo sapiens 9606 blood

SAMN30183380 T2DM-0034 T2DM-0034 Homo sapiens 9606 blood

SAMN30183381 T2DM-0035 T2DM-0035 Homo sapiens 9606 blood

SAMN30183382 T2DM-0036 T2DM-0036 Homo sapiens 9606 blood

SAMN30183383 T2DM-0037 T2DM-0037 Homo sapiens 9606 blood

SAMN30183384 T2DM-0038 T2DM-0038 Homo sapiens 9606 blood

SAMN30183385 T2DM-0039 T2DM-0039 Homo sapiens 9606 blood

SAMN30183386 T2DM-0040 T2DM-0040 Homo sapiens 9606 blood

SAMN30183387 T2DM-0041 T2DM-0041 Homo sapiens 9606 blood

SAMN30183388 T2DM-0042 T2DM-0042 Homo sapiens 9606 blood

SAMN30183389 T2DM-0043 T2DM-0043 Homo sapiens 9606 blood

SAMN30183390 T2DM-0044 T2DM-0044 Homo sapiens 9606 blood

SAMN30183391 T2DM-0045 T2DM-0045 Homo sapiens 9606 blood

SAMN30183392 T2DM-0046 T2DM-0046 Homo sapiens 9606 blood

SAMN30183393 T2DM-0047 T2DM-0047 Homo sapiens 9606 blood

SAMN30183394 T2DM-0048 T2DM-0048 Homo sapiens 9606 blood

SAMN30183395 T2DM-0049 T2DM-0049 Homo sapiens 9606 blood

SAMN30183396 T2DM-0050 T2DM-0050 Homo sapiens 9606 blood

SAMN30183397 T2DM-0051 T2DM-0051 Homo sapiens 9606 blood

SAMN30183398 T2DM-0052 T2DM-0052 Homo sapiens 9606 blood

SAMN30183399 T2DM-0053 T2DM-0053 Homo sapiens 9606 blood

SAMN30183400 T2DM-0054 T2DM-0054 Homo sapiens 9606 blood

SAMN30183401 T2DM-0055 T2DM-0055 Homo sapiens 9606 blood

SAMN30183402 T2DM-0056 T2DM-0056 Homo sapiens 9606 blood

SAMN30183403 T2DM-0057 T2DM-0057 Homo sapiens 9606 blood

SAMN30183404 T2DM-0058 T2DM-0058 Homo sapiens 9606 blood

SAMN30183405 T2DM-0059 T2DM-0059 Homo sapiens 9606 blood

SAMN30183406 T2DM-0060 T2DM-0060 Homo sapiens 9606 blood

SAMN30183407 T2DM-0061 T2DM-0061 Homo sapiens 9606 blood

SAMN30183408 T2DM-0062 T2DM-0062 Homo sapiens 9606 blood

SAMN30183409 T2DM-0063 T2DM-0063 Homo sapiens 9606 blood

SAMN30183410 T2DM-0064 T2DM-0064 Homo sapiens 9606 blood

SAMN30183411 T2DM-0065 T2DM-0065 Homo sapiens 9606 blood

SAMN30183412 T2DM-0066 T2DM-0066 Homo sapiens 9606 blood

SAMN30183413 T2DM-0067 T2DM-0067 Homo sapiens 9606 blood

SAMN30183414 T2DM-0068 T2DM-0068 Homo sapiens 9606 blood

SAMN30183415 T2DM-0069 T2DM-0069 Homo sapiens 9606 blood

SAMN30183416 T2DM-0070 T2DM-0070 Homo sapiens 9606 blood

SAMN30183417 T2DM-0071 T2DM-0071 Homo sapiens 9606 blood

SAMN30183418 T2DM-0072 T2DM-0072 Homo sapiens 9606 blood

SAMN30183419 T2DM-0073 T2DM-0073 Homo sapiens 9606 blood

SAMN30183420 T2DM-0074 T2DM-0074 Homo sapiens 9606 blood

SAMN30183421 T2DM-0075 T2DM-0075 Homo sapiens 9606 blood

SAMN30183422 T2DM-0076 T2DM-0076 Homo sapiens 9606 blood

SAMN30183423 T2DM-0077 T2DM-0077 Homo sapiens 9606 blood

SAMN30183424 T2DM-0078 T2DM-0078 Homo sapiens 9606 blood

SAMN30183425 T2DM-0079 T2DM-0079 Homo sapiens 9606 blood

SAMN30183426 T2DM-0080 T2DM-0080 Homo sapiens 9606 blood

SAMN30183427 T2DM-0081 T2DM-0081 Homo sapiens 9606 blood

SAMN30183428 T2DM-0082 T2DM-0082 Homo sapiens 9606 blood

SAMN30183429 T2DM-0083 T2DM-0083 Homo sapiens 9606 blood

SAMN30183430 T2DM-0084 T2DM-0084 Homo sapiens 9606 blood

SAMN30183431 T2DM-0085 T2DM-0085 Homo sapiens 9606 blood

SAMN30183432 T2DM-0086 T2DM-0086 Homo sapiens 9606 blood

SAMN30183433 T2DM-0087 T2DM-0087 Homo sapiens 9606 blood

SAMN30183434 T2DM-0088 T2DM-0088 Homo sapiens 9606 blood

SAMN30183435 T2DM-0089 T2DM-0089 Homo sapiens 9606 blood

SAMN30183436 T2DM-0090 T2DM-0090 Homo sapiens 9606 blood

SAMN30183437 T2DM-0091 T2DM-0091 Homo sapiens 9606 blood

SAMN30183438 T2DM-0092 T2DM-0092 Homo sapiens 9606 blood

SAMN30183439 T2DM-0093 T2DM-0093 Homo sapiens 9606 blood

SAMN30183440 T2DM-0094 T2DM-0094 Homo sapiens 9606 blood

SAMN30183441 T2DM-0095 T2DM-0095 Homo sapiens 9606 blood

SAMN30183442 T2DM-0096 T2DM-0096 Homo sapiens 9606 blood

SAMN30183443 T2DM-0097 T2DM-0097 Homo sapiens 9606 blood

SAMN30183444 T2DM-0098 T2DM-0098 Homo sapiens 9606 blood

SAMN30183445 T2DM-0099 T2DM-0099 Homo sapiens 9606 blood

SAMN30183446 T2DM-0100 T2DM-0100 Homo sapiens 9606 blood

SAMN30183447 T2DM-0101 T2DM-0101 Homo sapiens 9606 blood

SAMN30183448 T2DM-0102 T2DM-0102 Homo sapiens 9606 blood

SAMN30183449 T2DM-0103 T2DM-0103 Homo sapiens 9606 blood

SAMN30183450 T2DM-0104 T2DM-0104 Homo sapiens 9606 blood

SAMN30183451 T2DM-0105 T2DM-0105 Homo sapiens 9606 blood

SAMN30183452 T2DM-0106 T2DM-0106 Homo sapiens 9606 blood

SAMN30183453 T2DM-0107 T2DM-0107 Homo sapiens 9606 blood

SAMN30183454 T2DM-0108 T2DM-0108 Homo sapiens 9606 blood

SAMN30183455 T2DM-0109 T2DM-0109 Homo sapiens 9606 blood

SAMN30183456 T2DM-0110 T2DM-0110 Homo sapiens 9606 blood

SAMN30183457 T2DM-0111 T2DM-0111 Homo sapiens 9606 blood

SAMN30183458 T2DM-0112 T2DM-0112 Homo sapiens 9606 blood

SAMN30183459 T2DM-0113 T2DM-0113 Homo sapiens 9606 blood

SAMN30183460 T2DM-0114 T2DM-0114 Homo sapiens 9606 blood

SAMN30183461 T2DM-0115 T2DM-0115 Homo sapiens 9606 blood

SAMN30183462 T2DM-0116 T2DM-0116 Homo sapiens 9606 blood

SAMN30183463 T2DM-0117 T2DM-0117 Homo sapiens 9606 blood

SAMN30183464 T2DM-0118 T2DM-0118 Homo sapiens 9606 blood

SAMN30183465 T2DM-0119 T2DM-0119 Homo sapiens 9606 blood

SAMN30183466 T2DM-0120 T2DM-0120 Homo sapiens 9606 blood

SAMN30183467 T2DM-0121 T2DM-0121 Homo sapiens 9606 blood

SAMN30183468 T2DM-0122 T2DM-0122 Homo sapiens 9606 blood

SAMN30183469 T2DM-0123 T2DM-0123 Homo sapiens 9606 blood

SAMN30183470 T2DM-0124 T2DM-0124 Homo sapiens 9606 blood

SAMN30183471 T2DM-0125 T2DM-0125 Homo sapiens 9606 blood

SAMN30183472 T2DM-0126 T2DM-0126 Homo sapiens 9606 blood

SAMN30183473 T2DM-0127 T2DM-0127 Homo sapiens 9606 blood

SAMN30183474 T2DM-0128 T2DM-0128 Homo sapiens 9606 blood

SAMN30183475 T2DM-0129 T2DM-0129 Homo sapiens 9606 blood

SAMN30183476 T2DM-0130 T2DM-0130 Homo sapiens 9606 blood

SAMN30183477 T2DM-0131 T2DM-0131 Homo sapiens 9606 blood

SAMN30183478 T2DM-0132 T2DM-0132 Homo sapiens 9606 blood

SAMN30183479 T2DM-0133 T2DM-0133 Homo sapiens 9606 blood

SAMN30183480 T2DM-0134 T2DM-0134 Homo sapiens 9606 blood

SAMN30183481 T2DM-0135 T2DM-0135 Homo sapiens 9606 blood

SAMN30183482 T2DM-0136 T2DM-0136 Homo sapiens 9606 blood

SAMN30183483 T2DM-0137 T2DM-0137 Homo sapiens 9606 blood

SAMN30183484 T2DM-0138 T2DM-0138 Homo sapiens 9606 blood

SAMN30183485 T2DM-0139 T2DM-0139 Homo sapiens 9606 blood

SAMN30183486 T2DM-0140 T2DM-0140 Homo sapiens 9606 blood

SAMN30183487 T2DM-0141 T2DM-0141 Homo sapiens 9606 blood

SAMN30183488 T2DM-0142 T2DM-0142 Homo sapiens 9606 blood

SAMN30183489 T2DM-0143 T2DM-0143 Homo sapiens 9606 blood

SAMN30183490 T2DM-0144 T2DM-0144 Homo sapiens 9606 blood

SAMN30183491 T2DM-0145 T2DM-0145 Homo sapiens 9606 blood

SAMN30183492 T2DM-0146 T2DM-0146 Homo sapiens 9606 blood

SAMN30183493 T2DM-0147 T2DM-0147 Homo sapiens 9606 blood

SAMN30183494 T2DM-0148 T2DM-0148 Homo sapiens 9606 blood

SAMN30183495 T2DM-0149 T2DM-0149 Homo sapiens 9606 blood

SAMN30183496 T2DM-0150 T2DM-0150 Homo sapiens 9606 blood

SAMN30183497 T2DM-0151 T2DM-0151 Homo sapiens 9606 blood

SAMN30183498 T2DM-0152 T2DM-0152 Homo sapiens 9606 blood

SAMN30183499 T2DM-0153 T2DM-0153 Homo sapiens 9606 blood

SAMN30183500 T2DM-0154 T2DM-0154 Homo sapiens 9606 blood

SAMN30183501 T2DM-0155 T2DM-0155 Homo sapiens 9606 blood

SAMN30183502 T2DM-0156 T2DM-0156 Homo sapiens 9606 blood

SAMN30183503 T2DM-0157 T2DM-0157 Homo sapiens 9606 blood

SAMN30183504 T2DM-0158 T2DM-0158 Homo sapiens 9606 blood

SAMN30183505 T2DM-0159 T2DM-0159 Homo sapiens 9606 blood

SAMN30183506 T2DM-0160 T2DM-0160 Homo sapiens 9606 blood

SAMN30183507 T2DM-0161 T2DM-0161 Homo sapiens 9606 blood

SAMN30183508 T2DM-0162 T2DM-0162 Homo sapiens 9606 blood

SAMN30183509 T2DM-0163 T2DM-0163 Homo sapiens 9606 blood

SAMN30183510 T2DM-0164 T2DM-0164 Homo sapiens 9606 blood

SAMN30183511 T2DM-0165 T2DM-0165 Homo sapiens 9606 blood

SAMN30183512 T2DM-0166 T2DM-0166 Homo sapiens 9606 blood

SAMN30183513 T2DM-0167 T2DM-0167 Homo sapiens 9606 blood

SAMN30183514 T2DM-0168 T2DM-0168 Homo sapiens 9606 blood

SAMN30183515 T2DM-0169 T2DM-0169 Homo sapiens 9606 blood

SAMN30183516 T2DM-0170 T2DM-0170 Homo sapiens 9606 blood

SAMN30183517 T2DM-0171 T2DM-0171 Homo sapiens 9606 blood

SAMN30183518 T2DM-0172 T2DM-0172 Homo sapiens 9606 blood

SAMN30183519 T2DM-0173 T2DM-0173 Homo sapiens 9606 blood

SAMN30183520 T2DM-0174 T2DM-0174 Homo sapiens 9606 blood

SAMN30183521 T2DM-0175 T2DM-0175 Homo sapiens 9606 blood

SAMN30183522 T2DM-0176 T2DM-0176 Homo sapiens 9606 blood

SAMN30183523 T2DM-0177 T2DM-0177 Homo sapiens 9606 blood

SAMN30183524 T2DM-0178 T2DM-0178 Homo sapiens 9606 blood

SAMN30183525 T2DM-0179 T2DM-0179 Homo sapiens 9606 blood

SAMN30183526 T2DM-0180 T2DM-0180 Homo sapiens 9606 blood

SAMN30183527 T2DM-0181 T2DM-0181 Homo sapiens 9606 blood

SAMN30183528 T2DM-0182 T2DM-0182 Homo sapiens 9606 blood

SAMN30183529 T2DM-0183 T2DM-0183 Homo sapiens 9606 blood

SAMN30183530 T2DM-0184 T2DM-0184 Homo sapiens 9606 blood

SAMN30183531 T2DM-0185 T2DM-0185 Homo sapiens 9606 blood

SAMN30183532 T2DM-0186 T2DM-0186 Homo sapiens 9606 blood

SAMN30183533 T2DM-0187 T2DM-0187 Homo sapiens 9606 blood

SAMN30183534 T2DM-0188 T2DM-0188 Homo sapiens 9606 blood

SAMN30183535 T2DM-0189 T2DM-0189 Homo sapiens 9606 blood

SAMN30183536 T2DM-0190 T2DM-0190 Homo sapiens 9606 blood

SAMN30183537 T2DM-0191 T2DM-0191 Homo sapiens 9606 blood

SAMN30183538 T2DM-0192 T2DM-0192 Homo sapiens 9606 blood

SAMN30183539 T2DM-0193 T2DM-0193 Homo sapiens 9606 blood

SAMN30183540 T2DM-0194 T2DM-0194 Homo sapiens 9606 blood

SAMN30183541 T2DM-0195 T2DM-0195 Homo sapiens 9606 blood

SAMN30183542 T2DM-0196 T2DM-0196 Homo sapiens 9606 blood

SAMN30183543 T2DM-0197 T2DM-0197 Homo sapiens 9606 blood

SAMN30183544 T2DM-0198 T2DM-0198 Homo sapiens 9606 blood

SAMN30183545 T2DM-0199 T2DM-0199 Homo sapiens 9606 blood

SAMN30183546 T2DM-0200 T2DM-0200 Homo sapiens 9606 blood

SAMN30183547 T2DM-0201 T2DM-0201 Homo sapiens 9606 blood

SAMN30183548 T2DM-0202 T2DM-0202 Homo sapiens 9606 blood

SAMN30183549 T2DM-0203 T2DM-0203 Homo sapiens 9606 blood

SAMN30183550 T2DM-0204 T2DM-0204 Homo sapiens 9606 blood

SAMN30183551 T2DM-0205 T2DM-0205 Homo sapiens 9606 blood

SAMN30183552 T2DM-0206 T2DM-0206 Homo sapiens 9606 blood

SAMN30183553 T2DM-0207 T2DM-0207 Homo sapiens 9606 blood

SAMN30183554 T2DM-0208 T2DM-0208 Homo sapiens 9606 blood

SAMN30183555 T2DM-0209 T2DM-0209 Homo sapiens 9606 blood

SAMN30183556 T2DM-0210 T2DM-0210 Homo sapiens 9606 blood

SAMN30183557 T2DM-0211 T2DM-0211 Homo sapiens 9606 blood

SAMN30183558 T2DM-0212 T2DM-0212 Homo sapiens 9606 blood

SAMN30183559 T2DM-0213 T2DM-0213 Homo sapiens 9606 blood

SAMN30183560 T2DM-0214 T2DM-0214 Homo sapiens 9606 blood

SAMN30183561 T2DM-0215 T2DM-0215 Homo sapiens 9606 blood

SAMN30183562 T2DM-0216 T2DM-0216 Homo sapiens 9606 blood

SAMN30183563 T2DM-0217 T2DM-0217 Homo sapiens 9606 blood

SAMN30183564 T2DM-0218 T2DM-0218 Homo sapiens 9606 blood

SAMN30183565 T2DM-0219 T2DM-0219 Homo sapiens 9606 blood

SAMN30183566 T2DM-0220 T2DM-0220 Homo sapiens 9606 blood

SAMN30183567 T2DM-0221 T2DM-0221 Homo sapiens 9606 blood

SAMN30183568 T2DM-0222 T2DM-0222 Homo sapiens 9606 blood

SAMN30183569 T2DM-0223 T2DM-0223 Homo sapiens 9606 blood

SAMN30183570 T2DM-0224 T2DM-0224 Homo sapiens 9606 blood

SAMN30183571 T2DM-0225 T2DM-0225 Homo sapiens 9606 blood

SAMN30183572 T2DM-0226 T2DM-0226 Homo sapiens 9606 blood

SAMN30183573 T2DM-0227 T2DM-0227 Homo sapiens 9606 blood

SAMN30183574 T2DM-0228 T2DM-0228 Homo sapiens 9606 blood

SAMN30183575 T2DM-0229 T2DM-0229 Homo sapiens 9606 blood

SAMN30183576 T2DM-0230 T2DM-0230 Homo sapiens 9606 blood

SAMN30183577 T2DM-0231 T2DM-0231 Homo sapiens 9606 blood

SAMN30183578 T2DM-0232 T2DM-0232 Homo sapiens 9606 blood

SAMN30183579 T2DM-0233 T2DM-0233 Homo sapiens 9606 blood

SAMN30183580 T2DM-0234 T2DM-0234 Homo sapiens 9606 blood

SAMN30183581 T2DM-0235 T2DM-0235 Homo sapiens 9606 blood

SAMN30183582 T2DM-0236 T2DM-0236 Homo sapiens 9606 blood

SAMN30183583 T2DM-0237 T2DM-0237 Homo sapiens 9606 blood

SAMN30183584 T2DM-0238 T2DM-0238 Homo sapiens 9606 blood

SAMN30183585 T2DM-0239 T2DM-0239 Homo sapiens 9606 blood

SAMN30183586 T2DM-0240 T2DM-0240 Homo sapiens 9606 blood

SAMN30183587 T2DM-0241 T2DM-0241 Homo sapiens 9606 blood

SAMN30183588 T2DM-0242 T2DM-0242 Homo sapiens 9606 blood

SAMN30183589 T2DM-0243 T2DM-0243 Homo sapiens 9606 blood

SAMN30183590 T2DM-0244 T2DM-0244 Homo sapiens 9606 blood

SAMN30183591 T2DM-0245 T2DM-0245 Homo sapiens 9606 blood

SAMN30183592 T2DM-0246 T2DM-0246 Homo sapiens 9606 blood

SAMN30183593 T2DM-0247 T2DM-0247 Homo sapiens 9606 blood

SAMN30183594 T2DM-0248 T2DM-0248 Homo sapiens 9606 blood

SAMN30183595 T2DM-0249 T2DM-0249 Homo sapiens 9606 blood

SAMN30183596 T2DM-0250 T2DM-0250 Homo sapiens 9606 blood

SAMN30183597 T2DM-0251 T2DM-0251 Homo sapiens 9606 blood

SAMN30183598 T2DM-0252 T2DM-0252 Homo sapiens 9606 blood

SAMN30183599 T2DM-0253 T2DM-0253 Homo sapiens 9606 blood

SAMN30183600 T2DM-0254 T2DM-0254 Homo sapiens 9606 blood

SAMN30183601 T2DM-0255 T2DM-0255 Homo sapiens 9606 blood

SAMN30183602 T2DM-0256 T2DM-0256 Homo sapiens 9606 blood

SAMN30183603 T2DM-0257 T2DM-0257 Homo sapiens 9606 blood

SAMN30183604 T2DM-0258 T2DM-0258 Homo sapiens 9606 blood

SAMN30183605 T2DM-0259 T2DM-0259 Homo sapiens 9606 blood

SAMN30183606 T2DM-0260 T2DM-0260 Homo sapiens 9606 blood

SAMN30183607 T2DM-0261 T2DM-0261 Homo sapiens 9606 blood

SAMN30183608 T2DM-0262 T2DM-0262 Homo sapiens 9606 blood

SAMN30183609 T2DM-0263 T2DM-0263 Homo sapiens 9606 blood

SAMN30183610 T2DM-0264 T2DM-0264 Homo sapiens 9606 blood

SAMN30183611 T2DM-0265 T2DM-0265 Homo sapiens 9606 blood

SAMN30183612 T2DM-0266 T2DM-0266 Homo sapiens 9606 blood

SAMN30183613 T2DM-0267 T2DM-0267 Homo sapiens 9606 blood

SAMN30183614 T2DM-0268 T2DM-0268 Homo sapiens 9606 blood

SAMN30183615 T2DM-0269 T2DM-0269 Homo sapiens 9606 blood

SAMN30183616 T2DM-0270 T2DM-0270 Homo sapiens 9606 blood

SAMN30183617 T2DM-0271 T2DM-0271 Homo sapiens 9606 blood

SAMN30183618 T2DM-0272 T2DM-0272 Homo sapiens 9606 blood

SAMN30183619 T2DM-0273 T2DM-0273 Homo sapiens 9606 blood

SAMN30183620 T2DM-0274 T2DM-0274 Homo sapiens 9606 blood

SAMN30183621 T2DM-0275 T2DM-0275 Homo sapiens 9606 blood

SAMN30183622 T2DM-0276 T2DM-0276 Homo sapiens 9606 blood

SAMN30183623 T2DM-0277 T2DM-0277 Homo sapiens 9606 blood

SAMN30183624 T2DM-0278 T2DM-0278 Homo sapiens 9606 blood

SAMN30183625 T2DM-0279 T2DM-0279 Homo sapiens 9606 blood

SAMN30183626 T2DM-0280 T2DM-0280 Homo sapiens 9606 blood

SAMN30183627 T2DM-0281 T2DM-0281 Homo sapiens 9606 blood

SAMN30183628 T2DM-0282 T2DM-0282 Homo sapiens 9606 blood

SAMN30183629 T2DM-0283 T2DM-0283 Homo sapiens 9606 blood

SAMN30183630 T2DM-0284 T2DM-0284 Homo sapiens 9606 blood

SAMN30183631 T2DM-0285 T2DM-0285 Homo sapiens 9606 blood

SAMN30183632 T2DM-0286 T2DM-0286 Homo sapiens 9606 blood

SAMN30183633 T2DM-0287 T2DM-0287 Homo sapiens 9606 blood

SAMN30183634 T2DM-0288 T2DM-0288 Homo sapiens 9606 blood

SAMN30183635 T2DM-0289 T2DM-0289 Homo sapiens 9606 blood

SAMN30183636 T2DM-0290 T2DM-0290 Homo sapiens 9606 blood

SAMN30183637 T2DM-0291 T2DM-0291 Homo sapiens 9606 blood

SAMN30183638 T2DM-0292 T2DM-0292 Homo sapiens 9606 blood

SAMN30183639 T2DM-0293 T2DM-0293 Homo sapiens 9606 blood

SAMN30183640 T2DM-0294 T2DM-0294 Homo sapiens 9606 blood

SAMN30183641 T2DM-0295 T2DM-0295 Homo sapiens 9606 blood

SAMN30183642 T2DM-0296 T2DM-0296 Homo sapiens 9606 blood

SAMN30183643 T2DM-0297 T2DM-0297 Homo sapiens 9606 blood

SAMN30183644 T2DM-0298 T2DM-0298 Homo sapiens 9606 blood

SAMN30183645 T2DM-0299 T2DM-0299 Homo sapiens 9606 blood

SAMN30183646 T2DM-0300 T2DM-0300 Homo sapiens 9606 blood

SAMN30183647 control-0001 control-0001 Homo sapiens 9606 blood

SAMN30183648 control-0002 control-0002 Homo sapiens 9606 blood

SAMN30183649 control-0003 control-0003 Homo sapiens 9606 blood

SAMN30183650 control-0004 control-0004 Homo sapiens 9606 blood

SAMN30183651 control-0005 control-0005 Homo sapiens 9606 blood

SAMN30183652 control-0006 control-0006 Homo sapiens 9606 blood

SAMN30183653 control-0007 control-0007 Homo sapiens 9606 blood

SAMN30183654 control-0008 control-0008 Homo sapiens 9606 blood

SAMN30183655 control-0009 control-0009 Homo sapiens 9606 blood

SAMN30183656 control-0010 control-0010 Homo sapiens 9606 blood

SAMN30183657 control-0011 control-0011 Homo sapiens 9606 blood

SAMN30183658 control-0012 control-0012 Homo sapiens 9606 blood

SAMN30183659 control-0013 control-0013 Homo sapiens 9606 blood

SAMN30183660 control-0014 control-0014 Homo sapiens 9606 blood

SAMN30183661 control-0015 control-0015 Homo sapiens 9606 blood

SAMN30183662 control-0016 control-0016 Homo sapiens 9606 blood

SAMN30183663 control-0017 control-0017 Homo sapiens 9606 blood

SAMN30183664 control-0018 control-0018 Homo sapiens 9606 blood

SAMN30183665 control-0019 control-0019 Homo sapiens 9606 blood

SAMN30183666 control-0020 control-0020 Homo sapiens 9606 blood

SAMN30183667 control-0021 control-0021 Homo sapiens 9606 blood

SAMN30183668 control-0022 control-0022 Homo sapiens 9606 blood

SAMN30183669 control-0023 control-0023 Homo sapiens 9606 blood

SAMN30183670 control-0024 control-0024 Homo sapiens 9606 blood

SAMN30183671 control-0025 control-0025 Homo sapiens 9606 blood

SAMN30183672 control-0026 control-0026 Homo sapiens 9606 blood

SAMN30183673 control-0027 control-0027 Homo sapiens 9606 blood

SAMN30183674 control-0028 control-0028 Homo sapiens 9606 blood

SAMN30183675 control-0029 control-0029 Homo sapiens 9606 blood

SAMN30183676 control-0030 control-0030 Homo sapiens 9606 blood

SAMN30183677 control-0031 control-0031 Homo sapiens 9606 blood

SAMN30183678 control-0032 control-0032 Homo sapiens 9606 blood

SAMN30183679 control-0033 control-0033 Homo sapiens 9606 blood

SAMN30183680 control-0034 control-0034 Homo sapiens 9606 blood

SAMN30183681 control-0035 control-0035 Homo sapiens 9606 blood

SAMN30183682 control-0036 control-0036 Homo sapiens 9606 blood

SAMN30183683 control-0037 control-0037 Homo sapiens 9606 blood

SAMN30183684 control-0038 control-0038 Homo sapiens 9606 blood

SAMN30183685 control-0039 control-0039 Homo sapiens 9606 blood

SAMN30183686 control-0040 control-0040 Homo sapiens 9606 blood

SAMN30183687 control-0041 control-0041 Homo sapiens 9606 blood

SAMN30183688 control-0042 control-0042 Homo sapiens 9606 blood

SAMN30183689 control-0043 control-0043 Homo sapiens 9606 blood

SAMN30183690 control-0044 control-0044 Homo sapiens 9606 blood

SAMN30183691 control-0045 control-0045 Homo sapiens 9606 blood

SAMN30183692 control-0046 control-0046 Homo sapiens 9606 blood

SAMN30183693 control-0047 control-0047 Homo sapiens 9606 blood

SAMN30183694 control-0048 control-0048 Homo sapiens 9606 blood

SAMN30183695 control-0049 control-0049 Homo sapiens 9606 blood

SAMN30183696 control-0050 control-0050 Homo sapiens 9606 blood

SAMN30183697 control-0051 control-0051 Homo sapiens 9606 blood

SAMN30183698 control-0052 control-0052 Homo sapiens 9606 blood

SAMN30183699 control-0053 control-0053 Homo sapiens 9606 blood

SAMN30183700 control-0054 control-0054 Homo sapiens 9606 blood

SAMN30183701 control-0055 control-0055 Homo sapiens 9606 blood

SAMN30183702 control-0056 control-0056 Homo sapiens 9606 blood

SAMN30183703 control-0057 control-0057 Homo sapiens 9606 blood

SAMN30183704 control-0058 control-0058 Homo sapiens 9606 blood

SAMN30183705 control-0059 control-0059 Homo sapiens 9606 blood

SAMN30183706 control-0060 control-0060 Homo sapiens 9606 blood

SAMN30183707 control-0061 control-0061 Homo sapiens 9606 blood

SAMN30183708 control-0062 control-0062 Homo sapiens 9606 blood

SAMN30183709 control-0063 control-0063 Homo sapiens 9606 blood

SAMN30183710 control-0064 control-0064 Homo sapiens 9606 blood

SAMN30183711 control-0065 control-0065 Homo sapiens 9606 blood

SAMN30183712 control-0066 control-0066 Homo sapiens 9606 blood

SAMN30183713 control-0067 control-0067 Homo sapiens 9606 blood

SAMN30183714 control-0068 control-0068 Homo sapiens 9606 blood

SAMN30183715 control-0069 control-0069 Homo sapiens 9606 blood

SAMN30183716 control-0070 control-0070 Homo sapiens 9606 blood

SAMN30183717 control-0071 control-0071 Homo sapiens 9606 blood

SAMN30183718 control-0072 control-0072 Homo sapiens 9606 blood

SAMN30183719 control-0073 control-0073 Homo sapiens 9606 blood

SAMN30183720 control-0074 control-0074 Homo sapiens 9606 blood

SAMN30183721 control-0075 control-0075 Homo sapiens 9606 blood

SAMN30183722 control-0076 control-0076 Homo sapiens 9606 blood

SAMN30183723 control-0077 control-0077 Homo sapiens 9606 blood

SAMN30183724 control-0078 control-0078 Homo sapiens 9606 blood

SAMN30183725 control-0079 control-0079 Homo sapiens 9606 blood

SAMN30183726 control-0080 control-0080 Homo sapiens 9606 blood

SAMN30183727 control-0081 control-0081 Homo sapiens 9606 blood

SAMN30183728 control-0082 control-0082 Homo sapiens 9606 blood

SAMN30183729 control-0083 control-0083 Homo sapiens 9606 blood

SAMN30183730 control-0084 control-0084 Homo sapiens 9606 blood

SAMN30183731 control-0085 control-0085 Homo sapiens 9606 blood

SAMN30183732 control-0086 control-0086 Homo sapiens 9606 blood

SAMN30183733 control-0087 control-0087 Homo sapiens 9606 blood

SAMN30183734 control-0088 control-0088 Homo sapiens 9606 blood

SAMN30183735 control-0089 control-0089 Homo sapiens 9606 blood

SAMN30183736 control-0090 control-0090 Homo sapiens 9606 blood

SAMN30183737 control-0091 control-0091 Homo sapiens 9606 blood

SAMN30183738 control-0092 control-0092 Homo sapiens 9606 blood

SAMN30183739 control-0093 control-0093 Homo sapiens 9606 blood

SAMN30183740 control-0094 control-0094 Homo sapiens 9606 blood

SAMN30183741 control-0095 control-0095 Homo sapiens 9606 blood

SAMN30183742 control-0096 control-0096 Homo sapiens 9606 blood

SAMN30183743 control-0097 control-0097 Homo sapiens 9606 blood

SAMN30183744 control-0098 control-0098 Homo sapiens 9606 blood

SAMN30183745 control-0099 control-0099 Homo sapiens 9606 blood

SAMN30183746 control-0100 control-0100 Homo sapiens 9606 blood

SAMN30183747 control-0101 control-0101 Homo sapiens 9606 blood

SAMN30183748 control-0102 control-0102 Homo sapiens 9606 blood

SAMN30183749 control-0103 control-0103 Homo sapiens 9606 blood

SAMN30183750 control-0104 control-0104 Homo sapiens 9606 blood

SAMN30183751 control-0105 control-0105 Homo sapiens 9606 blood

SAMN30183752 control-0106 control-0106 Homo sapiens 9606 blood

SAMN30183753 control-0107 control-0107 Homo sapiens 9606 blood

SAMN30183754 control-0108 control-0108 Homo sapiens 9606 blood

SAMN30183755 control-0109 control-0109 Homo sapiens 9606 blood

SAMN30183756 control-0110 control-0110 Homo sapiens 9606 blood

SAMN30183757 control-0111 control-0111 Homo sapiens 9606 blood

SAMN30183758 control-0112 control-0112 Homo sapiens 9606 blood

SAMN30183759 control-0113 control-0113 Homo sapiens 9606 blood

SAMN30183760 control-0114 control-0114 Homo sapiens 9606 blood

SAMN30183761 control-0115 control-0115 Homo sapiens 9606 blood

SAMN30183762 control-0116 control-0116 Homo sapiens 9606 blood

SAMN30183763 control-0117 control-0117 Homo sapiens 9606 blood

SAMN30183764 control-0118 control-0118 Homo sapiens 9606 blood

SAMN30183765 control-0119 control-0119 Homo sapiens 9606 blood

SAMN30183766 control-0120 control-0120 Homo sapiens 9606 blood

SAMN30183767 control-0121 control-0121 Homo sapiens 9606 blood

SAMN30183768 control-0122 control-0122 Homo sapiens 9606 blood

SAMN30183769 control-0123 control-0123 Homo sapiens 9606 blood

SAMN30183770 control-0124 control-0124 Homo sapiens 9606 blood

SAMN30183771 control-0125 control-0125 Homo sapiens 9606 blood

SAMN30183772 control-0126 control-0126 Homo sapiens 9606 blood

SAMN30183773 control-0127 control-0127 Homo sapiens 9606 blood

SAMN30183774 control-0128 control-0128 Homo sapiens 9606 blood

SAMN30183775 control-0129 control-0129 Homo sapiens 9606 blood

SAMN30183776 control-0130 control-0130 Homo sapiens 9606 blood

SAMN30183777 control-0131 control-0131 Homo sapiens 9606 blood

SAMN30183778 control-0132 control-0132 Homo sapiens 9606 blood

SAMN30183779 control-0133 control-0133 Homo sapiens 9606 blood

SAMN30183780 control-0134 control-0134 Homo sapiens 9606 blood

SAMN30183781 control-0135 control-0135 Homo sapiens 9606 blood

SAMN30183782 control-0136 control-0136 Homo sapiens 9606 blood

SAMN30183783 control-0137 control-0137 Homo sapiens 9606 blood

SAMN30183784 control-0138 control-0138 Homo sapiens 9606 blood

SAMN30183785 control-0139 control-0139 Homo sapiens 9606 blood

SAMN30183786 control-0140 control-0140 Homo sapiens 9606 blood

SAMN30183787 control-0141 control-0141 Homo sapiens 9606 blood

SAMN30183788 control-0142 control-0142 Homo sapiens 9606 blood

SAMN30183789 control-0143 control-0143 Homo sapiens 9606 blood

SAMN30183790 control-0144 control-0144 Homo sapiens 9606 blood

SAMN30183791 control-0145 control-0145 Homo sapiens 9606 blood

SAMN30183792 control-0146 control-0146 Homo sapiens 9606 blood

SAMN30183793 control-0147 control-0147 Homo sapiens 9606 blood

SAMN30183794 control-0148 control-0148 Homo sapiens 9606 blood

SAMN30183795 control-0149 control-0149 Homo sapiens 9606 blood

SAMN30183796 control-0150 control-0150 Homo sapiens 9606 blood

SAMN30183797 control-0151 control-0151 Homo sapiens 9606 blood

SAMN30183798 control-0152 control-0152 Homo sapiens 9606 blood

SAMN30183799 control-0153 control-0153 Homo sapiens 9606 blood

SAMN30183800 control-0154 control-0154 Homo sapiens 9606 blood

SAMN30183801 control-0155 control-0155 Homo sapiens 9606 blood

SAMN30183802 control-0156 control-0156 Homo sapiens 9606 blood

SAMN30183803 control-0157 control-0157 Homo sapiens 9606 blood

SAMN30183804 control-0158 control-0158 Homo sapiens 9606 blood

SAMN30183805 control-0159 control-0159 Homo sapiens 9606 blood

SAMN30183806 control-0160 control-0160 Homo sapiens 9606 blood

SAMN30183807 control-0161 control-0161 Homo sapiens 9606 blood

SAMN30183808 control-0162 control-0162 Homo sapiens 9606 blood

SAMN30183809 control-0163 control-0163 Homo sapiens 9606 blood

SAMN30183810 control-0164 control-0164 Homo sapiens 9606 blood

SAMN30183811 control-0165 control-0165 Homo sapiens 9606 blood

SAMN30183812 control-0166 control-0166 Homo sapiens 9606 blood

SAMN30183813 control-0167 control-0167 Homo sapiens 9606 blood

SAMN30183814 control-0168 control-0168 Homo sapiens 9606 blood

SAMN30183815 control-0169 control-0169 Homo sapiens 9606 blood

SAMN30183816 control-0170 control-0170 Homo sapiens 9606 blood

SAMN30183817 control-0171 control-0171 Homo sapiens 9606 blood

SAMN30183818 control-0172 control-0172 Homo sapiens 9606 blood

SAMN30183819 control-0173 control-0173 Homo sapiens 9606 blood

SAMN30183820 control-0174 control-0174 Homo sapiens 9606 blood

SAMN30183821 control-0175 control-0175 Homo sapiens 9606 blood

SAMN30183822 control-0176 control-0176 Homo sapiens 9606 blood

SAMN30183823 control-0177 control-0177 Homo sapiens 9606 blood

SAMN30183824 control-0178 control-0178 Homo sapiens 9606 blood

SAMN30183825 control-0179 control-0179 Homo sapiens 9606 blood

SAMN30183826 control-0180 control-0180 Homo sapiens 9606 blood

SAMN30183827 control-0181 control-0181 Homo sapiens 9606 blood

SAMN30183828 control-0182 control-0182 Homo sapiens 9606 blood

SAMN30183829 control-0183 control-0183 Homo sapiens 9606 blood

SAMN30183830 control-0184 control-0184 Homo sapiens 9606 blood

SAMN30183831 control-0185 control-0185 Homo sapiens 9606 blood

SAMN30183832 control-0186 control-0186 Homo sapiens 9606 blood

SAMN30183833 control-0187 control-0187 Homo sapiens 9606 blood

SAMN30183834 control-0188 control-0188 Homo sapiens 9606 blood

SAMN30183835 control-0189 control-0189 Homo sapiens 9606 blood

SAMN30183836 control-0190 control-0190 Homo sapiens 9606 blood

SAMN30183837 control-0191 control-0191 Homo sapiens 9606 blood

SAMN30183838 control-0192 control-0192 Homo sapiens 9606 blood

SAMN30183839 control-0193 control-0193 Homo sapiens 9606 blood

SAMN30183840 control-0194 control-0194 Homo sapiens 9606 blood

SAMN30183841 control-0195 control-0195 Homo sapiens 9606 blood

SAMN30183842 control-0196 control-0196 Homo sapiens 9606 blood

SAMN30183843 control-0197 control-0197 Homo sapiens 9606 blood

SAMN30183844 control-0198 control-0198 Homo sapiens 9606 blood

SAMN30183845 control-0199 control-0199 Homo sapiens 9606 blood

SAMN30183846 control-0200 control-0200 Homo sapiens 9606 blood
