# Supplementary material for: Effects of PPARD gene variants on the therapeutic responses to exenatide in chinese patients with type 2 diabetes mellitus
Source: Front Endocrinol (Lausanne). 2022 Aug 16;13:949990. doi: 10.3389/fendo.2022.949990 (PMC9424689; doi:10.3389/fendo.2022.949990)
Supplement: Supplementary file 2 [file Table_1.docx]

**Table S1** Clinical characteristics of patients with T2DM before and after exenatide treatment (n=105).

| **Parameters** | **Group** | | | **Overall**  ***P* value** | **Adjusted *P* value** | | |
| --- | --- | --- | --- | --- | --- | --- | --- |
|  | **Baseline** | **3 months** | **6 months** |  | **Baseline to 3 months** | **3 to 6 months** | **Baseline to 6 months** |
| BMI (kg/m^2^) | 29.13±3.36 | 28.33±3.14 | 27.97±3.12 | 0.000 | 0.000 | 0.000 | 0.000 |
| WHR | 0.97±0.06 | 0.95±0.05 | 0.94±0.05 | 0.000 | 0.000 | 0.000 | 0.000 |
| FPG (mmol/L) | 10.35±2.60 | 7.99±1.69 | 7.95±2.24 | 0.000 | 0.000 | 0.864 | 0.000 |
| PPG (mmol/L) | 14.04±3.82 | 10.46±2.25 | 9.98±2.16 | 0.000 | 0.000 | 0.018 | 0.000 |
| HbA1c (%) | 9.80±1.17 | 8.29±1.11 | 8.20±1.23 | 0.000 | 0.000 | 0.463 | 0.000 |
| FINS (mU/L) | 13.73±6.84 | 15.33±7.53 | 15.92±6.99 | 0.003 | 0.007 | 0.027 | 0.001 |
| PINS (mU/L) | 35.57±23.01 | 36.62±22.92 | 39.22±25.22 | 0.199 | 0.526 | 0.103 | 0.097 |
| HOMA-IR | 6.18±3.43 | 5.29±2.86 | 5.59±3.01 | 0.017 | 0.002 | 0.269 | 0.122 |
| HOMA-B | 48.83±36.16 | 81.88±48.97 | 84.46±52.08 | 0.000 | 0.000 | 0.537 | 0.000 |
| TG (mmol/L) | 2.61±2.17 | 1.96±1.24 | 2.05±1.38 | 0.002 | 0.000 | 0.438 | 0.005 |
| TC (mmol/L) | 5.16±1.37 | 4.53±0.95 | 4.44±1.09 | 0.000 | 0.000 | 0.241 | 0.000 |
| HDL-C (mmol/L) | 1.10±0.26 | 1.12±0.24 | 1.13±0.24 | 0.329 | 0.261 | 0.530 | 0.140 |
| LDL-C (mmol/L) | 2.95±0.91 | 2.57±0.74 | 2.47±0.86 | 0.000 | 0.000 | 0.116 | 0.000 |

BMI = body mass index; WHR = waist to hip ratio; FPG = fasting plasma glucose; PPG = postprandial plasma glucose; HbA_1c_ = hemoglobin A_1c_; FINS = fasting serum insulin; PINS = postprandial serum insulin; HOMA-IR = homeostasis model assessment for insulin resistance; HOMA-B = homeostasis model assessment for beta cell function; TG = triglyceride; TC = total cholesterol; HDL-C = high-density lipoprotein-cholesterol; and LDL-C = low-density lipoprotein-cholesterol.

**Table S2** Comparisons of differential values (DV) in patients with T2DM with different *PPARD* genotypes before and after exenatide treatment.

| Parameters | *PPARD* rs2016520 | | ^a^*P* value | *PPARD* rs3777744 | | ^b^*P* value |
| --- | --- | --- | --- | --- | --- | --- |
|  | TT | TC+CC |  | AA | AG+GG |  |
| N (male/female) | 62 (40/22) | 43 (33/10) | 0.181 | 50 (35/15) | 55 (38/17) | 0.919 |
| Age (years) | 45.69±13.93 | 46.77±12.25 | 0.684 | 46.46±12.02 | 45.84±14.33 | 0.811 |
| BMI (kg/m^2^) |  |  |  |  |  |  |
| Baseline | 29.30±3.53 | 28.89±3.13 | 0.544 | 28.84±3.38 | 29.40±3.36 | 0.400 |
| 3 months | 28.49±3.26 | 28.10±2.97 | 0.531 | 28.11±3.12 | 28.52±3.17 | 0.508 |
| 6 months | 28.07±3.28 | 27.82±2.92 | 0.692 | 27.69±3.14 | 28.23±3.11 | 0.375 |
| WHR |  |  |  |  |  |  |
| Baseline | 0.97±0.05 | 0.97±0.06 | 0.830 | 0.97±0.06 | 0.97±0.05 | 0.899 |
| 3 months | 0.95±0.05 | 0.95±0.05 | 0.716 | 0.95±0.06 | 0.95±0.05 | 0.676 |
| 6 months | 0.94±0.07 | 0.94±0.08 | 0.800 | 0.92±0.08 | 0.96±0.07 | 0.016 |
| FPG (mmol/L) |  |  |  |  |  |  |
| Baseline | 10.49±2.77 | 10.15±2.35 | 0.520 | 10.19±2.66 | 10.50±2.56 | 0.545 |
| 3 months | 7.88±1.72 | 8.14±1.64 | 0.433 | 7.83±1.36 | 8.13±1.94 | 0.356 |
| 6 months | 8.04±2.26 | 7.82±2.23 | 0.635 | 7.35±1.49 | 8.50±2.65 | 0.008 |
| PPG (mmol/L) |  |  |  |  |  |  |
| Baseline | 13.84±3.85 | 14.34±3.80 | 0.512 | 14.07±4.31 | 14.01±3.35 | 0.937 |
| 3 months | 10.34±2.29 | 10.63±2.19 | 0.514 | 10.26±2.12 | 10.64±2.36 | 0.381 |
| 6 months | 9.84±2.22 | 10.18±2.09 | 0.430 | 9.80±2.04 | 10.14±2.27 | 0.429 |
| HbA1c (%) |  |  |  |  |  |  |
| Baseline | 9.80±1.17 | 9.81±1.18 | 0.960 | 9.66±1.12 | 9.93±1.20 | 0.222 |
| 3 months | 8.31±1.15 | 8.25±1.07 | 0.778 | 8.06±0.93 | 8.50±1.23 | 0.042 |
| 6 months | 8.20±1.25 | 8.20±1.23 | 0.962 | 7.95±1.03 | 8.63±1.36 | 0.005 |
| FINS (mU/L) |  |  |  |  |  |  |
| Baseline | 14.42±7.12 | 12.73±6.36 | 0.215 | 13.44±6.09 | 13.98±7.51 | 0.689 |
| 3 months | 15.83±7.34 | 14.62±7.82 | 0.423 | 15.13±6.64 | 15.52±8.31 | 0.792 |
| 6 months | 17.32±7.28 | 13.89±6.08 | 0.013 | 15.01±6.08 | 16.75±7.69 | 0.204 |
| PINS (mU/L) |  |  |  |  |  |  |
| Baseline | 39.25±25.07 | 30.27±18.72 | 0.049 | 34.84±21.67 | 36.24±24.35 | 0.757 |
| 3 months | 38.51±24.20 | 33.90±20.91 | 0.313 | 36.08±22.25 | 37.11±23.70 | 0.819 |
| 6 months | 43.35±25.24 | 33.27±24.26 | 0.043 | 38.82±24.69 | 39.59±25.92 | 0.877 |
| HOMA-IR |  |  |  |  |  |  |
| Baseline | 6.55±3.72 | 5.65±2.93 | 0.184 | 6.01±3.20 | 6.33±3.65 | 0.628 |
| 3 months | 5.37±2.86 | 5.17±2.89 | 0.727 | 5.06±2.46 | 5.49±3.18 | 0.450 |
| 6 months | 6.21±3.02 | 4.70±2.78 | 0.011 | 4.84±2.29 | 6.27±3.41 | 0.014 |
| HOMA-B |  |  |  |  |  |  |
| Baseline | 50.90±36.90 | 45.83±35.28 | 0.483 | 48.92±33.27 | 48.75±38.91 | 0.981 |
| 3 months | 88.41±51.75 | 72.47±43.53 | 0.101 | 84.18±44.21 | 79.79±53.25 | 0.648 |
| 6 months | 89.54±51.00 | 77.12±53.34 | 0.231 | 88.65±50.13 | 80.64±53.97 | 0.434 |
| TC (mmol/L) |  |  |  |  |  |  |
| Baseline | 5.30±1.41 | 4.97±1.31 | 0.233 | 5.08±1.53 | 5.24±1.23 | 0.559 |
| 3 months | 4.57±0.96 | 4.46±0.95 | 0.552 | 4.52±0.96 | 4.53±0.95 | 0.901 |
| 6 months | 4.53±1.22 | 4.31±0.85 | 0.320 | 4.35±0.91 | 4.52±1.23 | 0.420 |
| TG (mmol/L) |  |  |  |  |  |  |
| Baseline | 2.89±2.03 | 2.21±2.33 | 0.118 | 2.44±2.01 | 2.77±2.31 | 0.433 |
| 3 months | 2.20±1.44 | 1.62±0.79 | 0.018 | 1.87±1.29 | 2.04±1.21 | 0.497 |
| 6 months | 2.13±1.19 | 1.95±1.63 | 0.507 | 1.88±1.16 | 2.21±1.54 | 0.229 |
| HDL-C (mmol/L) |  |  |  |  |  |  |
| Baseline | 1.10±0.23 | 1.10±0.31 | 0.988 | 1.10±0.30 | 1.09±0.22 | 0.888 |
| 3 months | 1.11±0.20 | 1.13±0.29 | 0.740 | 1.15±0.29 | 1.10±0.19 | 0.279 |
| 6 months | 1.15±0.21 | 1.11±0.28 | 0.351 | 1.13±0.25 | 1.13±0.23 | 0.918 |
| LDL-C (mmol/L) |  |  |  |  |  |  |
| Baseline | 3.03±1.01 | 2.85±0.75 | 0.335 | 2.91±0.88 | 2.99±0.95 | 0.643 |
| 3 months | 2.52±0.74 | 2.63±0.74 | 0.498 | 2.54±0.73 | 2.60±0.75 | 0.705 |
| 6 months | 2.52±0.99 | 2.39±0.65 | 0.428 | 2.38±0.75 | 2.54±0.96 | 0.341 |

BMI = body mass index; WHR = waist to hip ratio; FPG = fasting plasma glucose; PPG = postprandial plasma glucose; HbA1c = hemoglobin A1c; FINS = fasting serum insulin; PINS = postprandial serum insulin; HOMA-IR = homeostasis model assessment for insulin resistance; HOMA-B = homeostasis model assessment for beta cell function; TG = triglyceride; TC = total cholesterol; HDL-C = high-density lipoprotein-cholesterol; and LDL-C = low-density lipoprotein-cholesterol.

^a^Dominant model: TT vs. (TC + CC).

^b^Dominant model: AA vs. (AG + GG).
