# Supplementary figures and images for: Effects of PPARD gene variants on the therapeutic responses to exenatide in chinese patients with type 2 diabetes mellitus
Source: Front Endocrinol (Lausanne). 2022 Aug 16;13:949990. doi: 10.3389/fendo.2022.949990 (PMC9424689; doi:10.3389/fendo.2022.949990)

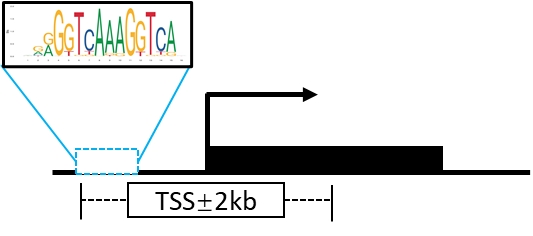

Supplement: Supplementary Figure 1 — Predicted GLP-1R binding motif site sequence from the database JASPAR. [file Image_1.jpeg]

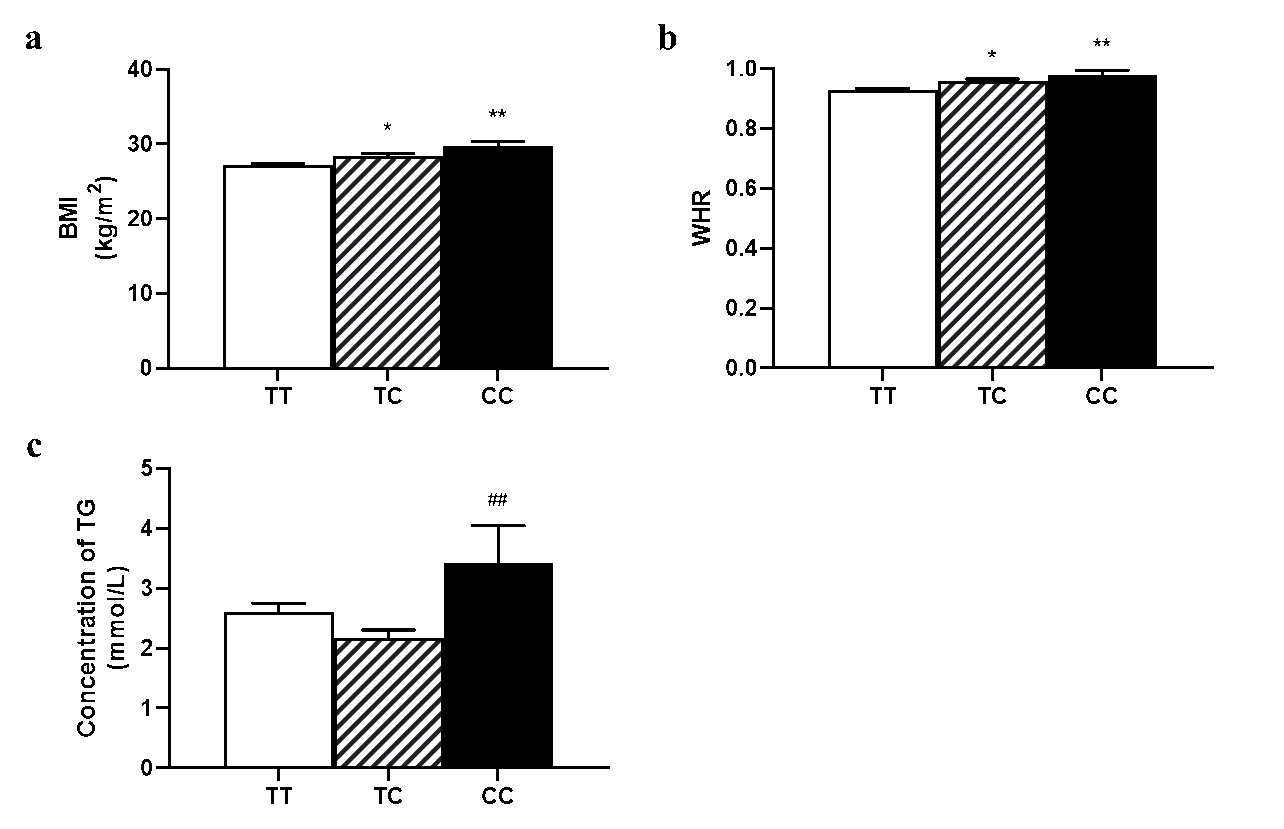

Supplement: Supplementary Figure 2 — Baseline levels of body mass index (BMI) (A), waist to hip ratio (WHR) (B) and triglyceride (TG) (C) in T2DM patients with TT (n = 148), TC (n = 125) and CC (n = 27) genotypes of PPARD rs22016520. *P < 0.05 and **P<0.01 compared with the TT genotype group, ## P<0.01 compared with the TC genotype group (n = 300). [file Image_2.jpeg]

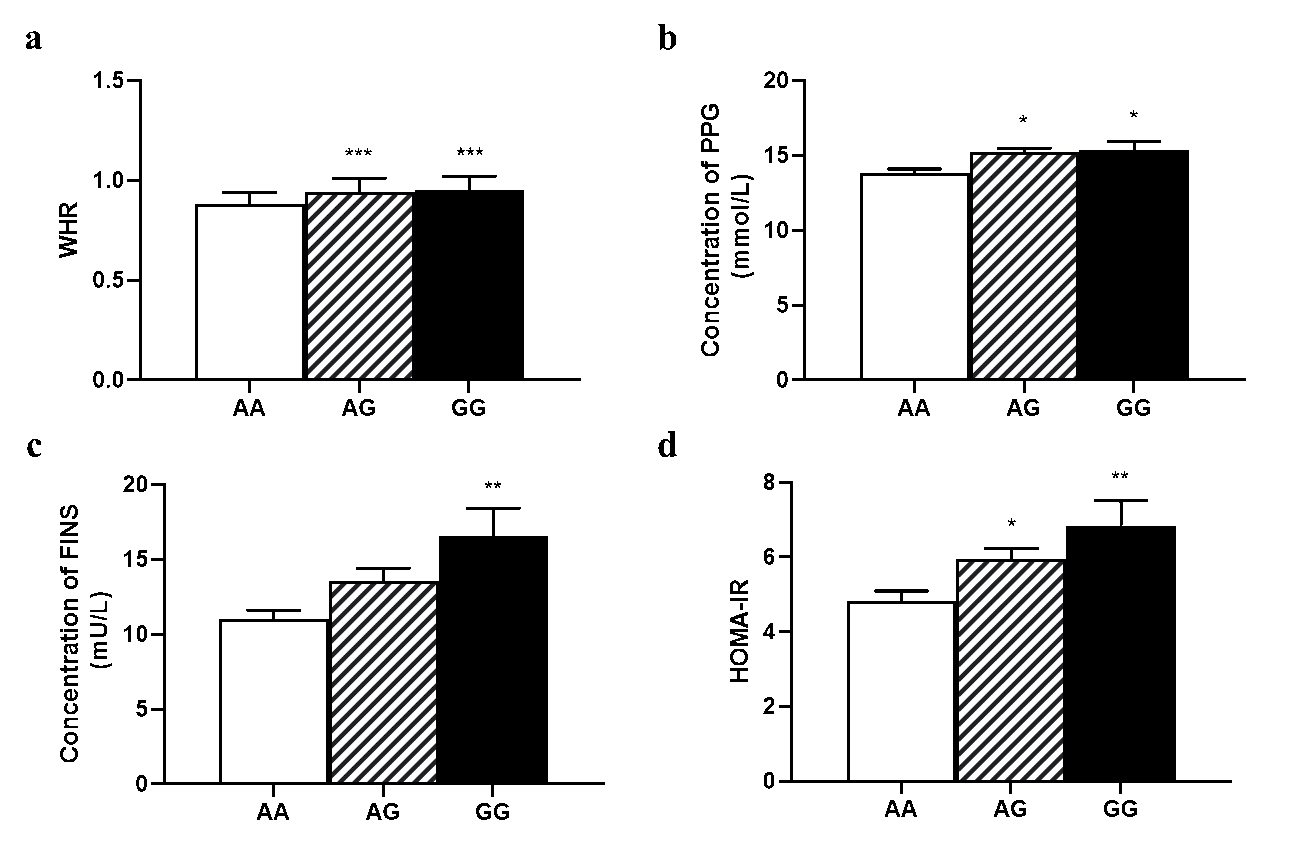

Supplement: Supplementary Figure 3 — Baseline levels of waist to hip ratio (WHR) (A), postprandial plasma glucose (PPG) (B), fasting serum insulin (FINS) (C) and homeostasis model assessment for insulin resistance (HOMA-IR) (D) in T2DM patients with AA (n = 95), AG (n = 150) or GG (n = 55) genotypes of PPARD rs3777744. *P < 0.05, **P < 0.01 and ***P < 0.001compared with the AA genotype group (n = 300). [file Image_3.jpeg]

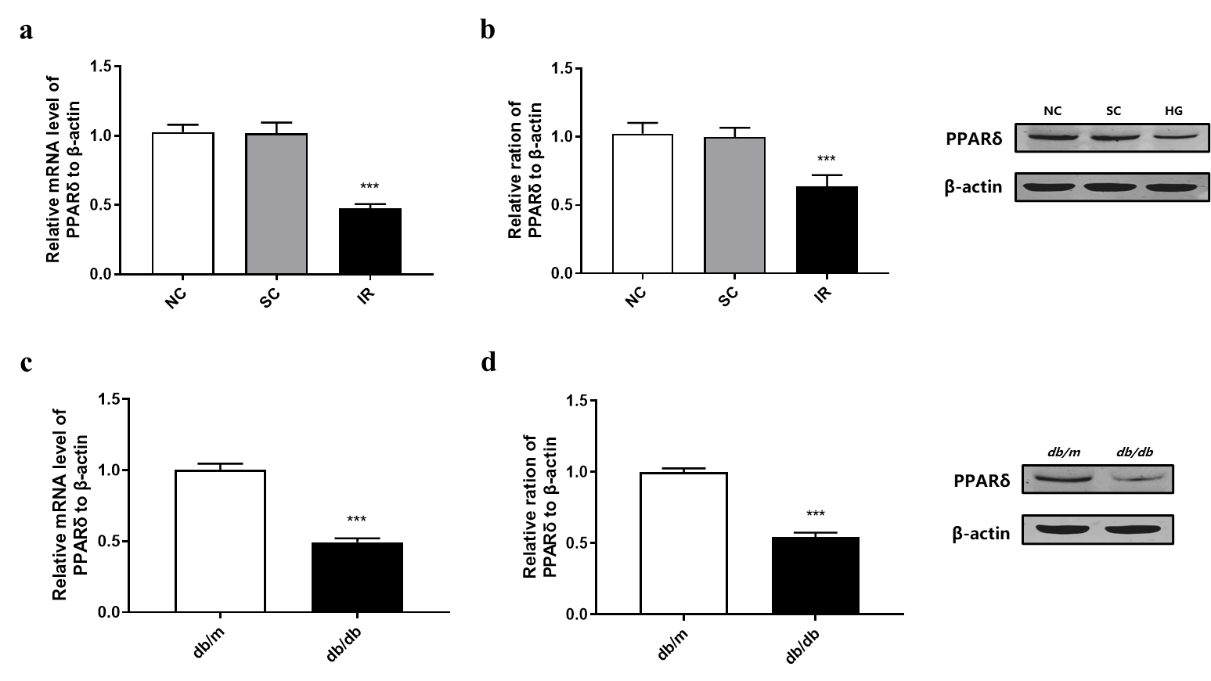

Supplement: Supplementary Figure 4 — The expression of PPARδ decreased in IR HepG2 cell model and db/db mice. (A) The mRNA level of PPARδ in HepG2 cells was measured by RT-PCR. Data are expressed as the mean ± SE, n = 3. (B) The relative protein expression level of PPARδ in HepG2 cells was measured by Western blot. Data are expressed as the mean ± SE, n = 3. *** P < 0.001 compared with NC. (C) The mRNA level of PPARδ in db/db mice was measured by RT-PCR. Data are expressed as the mean ± SE, n = 6. (D) The relative protein expression level of PPARδ in db/db mice was measured by Western blot. Data are expressed as the mean ± SE, n = 6. *** P < 0.001 compared with db/m. [file Image_4.jpeg]

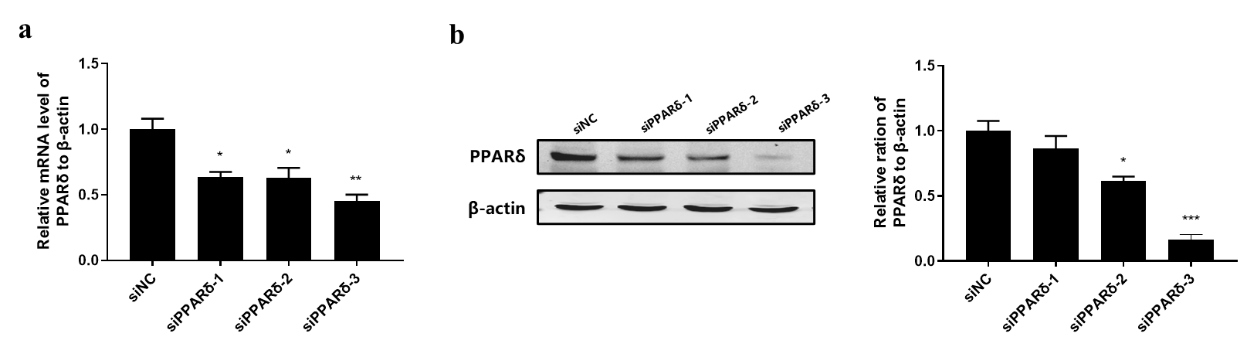

Supplement: Supplementary Figure 5 — Validation of PPARδ plasmid knockdown efficiency in HepG2 cells. (A) The mRNA level of PPARδ in HepG2 cells was measured by RT-PCR. (B) The relative protein expression level of PPARδ in HepG2 cells was measured by Western blot. Data are expressed as the mean ± SE, n = 3. * P < 0.05, ** P < 0.01 and *** P < 0.001 compared with siNC. [file Image_5.jpeg]
